# Recurrence relations for orthogonal polynomials for PDEs in polar and cylindrical geometries

**DOI:** 10.1186/s40064-016-3217-y

**Published:** 2016-09-15

**Authors:** Megan Richardson, James V. Lambers

**Affiliations:** Department of Mathematics, The University of Southern Mississippi, 118 College Dr #5045, Hattiesburg, MS 39406 USA

**Keywords:** Spectral-Galerkin, Polar coordinates, Legendre polynomials

## Abstract

This paper introduces two families of orthogonal polynomials on the interval (−1,1), with weight function $\omega (x)\equiv 1$. The first family satisfies the boundary condition $p(1)=0$, and the second one satisfies the boundary conditions $p(-1)=p(1)=0$. These boundary conditions arise naturally from PDEs defined on a disk with Dirichlet boundary conditions and the requirement of regularity in Cartesian coordinates. The families of orthogonal polynomials are obtained by orthogonalizing short linear combinations of Legendre polynomials that satisfy the same boundary conditions. Then, the three-term recurrence relations are derived. Finally, it is shown that from these recurrence relations, one can efficiently compute the corresponding recurrences for generalized Jacobi polynomials that satisfy the same boundary conditions.

## Background

When mapping PDEs to polar or cylindrical geometries to rectangular domains using polar coordinates, it makes sense to use spectral methods (Shen 1997). Numerous algorithms based on spectral-collocation and spectral-tau methods already exist. See, for example, Canuto et al. (1987), Eisen et al. (1991), Fornberg (1995), Gottlieb and Orszag (1977), Huang and Sloan (1993).

After applying separation of variables in polar coordinates, the resulting PDEs that depend on the radial coordinate *r* and time *t* can be solved numerically using a Legendre-Galerkin formulation similar to that used for the steady-state problem (Shen 1997). It is natural to use bases of polynomials that satisfy the boundary conditions for each PDE, and these can easily be obtained by taking short linear combinations of Legendre polynomials.

Unlike Legendre polynomials, the bases used in Shen (1997) are not orthogonal with respect to the weight function $\omega (x)\equiv 1$. In Shen (2003) orthogonal bases were introduced that also satisfy these same boundary conditions. They are generalized Jacobi polynomials (GJPs) with indices $\alpha ,\beta \le -1$, orthogonal with respect to the weight function $\omega ^{\alpha ,\beta }(x)\equiv (1-x)^\alpha (1+x)^\beta$. GJPs corresponding to specific indices $(\alpha ,\beta )$ were introduced in Shen (2003) for the purpose of solving differential equations of odd higher order. Generalization to other (non-integer) indices was carried out in Guo et al. (2009) to obtain families of orthogonal polynomials for Chebyshev spectral methods or problems with singular coefficients. However, although these GJPs can be described in terms of short linear combinations of Legendre polynomials, at least for certain index pairs of interest (Guo et al. 2009; Shen 2003), the three-term recurrence relations characteristic of families of orthogonal polynomials have not been developed in these cases.

In this paper, we use the bases from Shen (1997) to develop families of polynomials that are orthogonal with respect to $\omega (x)\equiv 1$ and satisfy the requisite boundary conditions, to facilitate transformation between physical and frequency space without using functions such as the Legendre polynomials that lie outside of the solution space. These families can also be efficiently modified to work with alternative weight functions, thus leading to the development of new numerical methods. In particular, it is demonstrated that these new families can be used to obtain three-term recurrence relations for the GJPs that satisfy the same boundary conditions.

The outline of the paper is as follows. In section “Variational formulation”, we provide context for these families of polynomials by adapting the variational formulation employed in Shen (1997) to the time-dependent PDE (1)–(3). In section “The case *m* = 0” we develop orthogonal polynomials with unit weight function satisfying the boundary conditions $p(1)=0$. In section “The case *m* ≠ 0” we do the same for the boundary conditions $p(-1)=p(1)=0$. In section “Recurrence relations for generalized jacobi polynomials” we describe how these families of orthogonal polynomials can be efficiently modified to obtain three-term recurrence relations for GJPs as described in Guo et al. (2009), Shen (2003). Concluding remarks and directions for future work are given in section “Conclusions”.

## Variational formulation

In this section, we describe one possible context in which the sequences of orthogonal polynomials discussed in this paper can be applied.

### Conversion to polar coordinates

We consider the reaction-diffusion equation on a unit disk

1 $$\begin{aligned} \Delta U-\alpha U & =  \frac{\partial U}{\partial t} \quad \mathrm {in\,\,}\varOmega =\left\{ \left( x,y\right) :\, x^{2}+y^{2}<1\right\},\quad t>0 \end{aligned}$$

2 $$\begin{aligned} U & = 0 \quad \mathrm {on} \quad \partial \varOmega ,\end{aligned}$$

3 $$\begin{aligned} U(x,y,0) & =  F(x,y) \quad \mathrm {on} \;\varOmega , \end{aligned}$$

where $\alpha$ is a constant.

Following the approach used in Shen (1997) for a steady-state problem, we can convert the IBVP in (1)–(3) to polar coordinates by applying the polar transformation $$x=r\cos \theta ,$$$$y=r\sin \theta $$ and letting $$u\left( r,\theta \right) =U\left( r\cos \theta ,r\sin \theta \right) ,$$$$f\left( r,\theta \right) =F\left( r\cos \theta ,r\sin \theta \right) .$$ The resulting problem in polar coordinates is as follows:

4 $$\begin{aligned} u_{rr}+\frac{1}{r}u_{r}+\frac{1}{r^{2}}u_{\theta \theta }-\alpha u & = \frac{\partial u}{\partial t}, \quad \left( r,\theta \right) \in Q=\left( 0,1\right) \times [0,2\pi ),\\ u\left( 1,t\right) & =  0,\quad \theta \in [0,2\pi ),\quad u  \text{ is } 2\pi \text{-periodic } \mathrm {in}  \, \theta ,\nonumber \\ u(r,\theta ,0) & = \frac{\partial u}{\partial t}. \nonumber \end{aligned}$$

The solution is represented using the Fourier series

5 $$\begin{aligned} u\left( r,t\right) =\sum _{\left| {m}\right| =0}^{\infty } \left[ u_{1,m}(r,t)\cos (m\theta )+u_{2,m}(r,t)\sin (m\theta )\right] . \end{aligned}$$

The Fourier coefficients $u_{1,m}(r,t)$, $u_{2,m}(r,t)$ must satisfy the boundary conditions $u_{1,m}(1,t) = u_{2,m}(1,t) = 0$ for $m=0,1,2,\ldots$ Due to the singularity at the pole $r=0,$ we must impose additional pole conditions on (5) to have regularity in Cartesian coordinates. For $u(r,\theta ,t)$ to be infinitely differentiable in the Cartesian plane, the additional pole conditions are Shen (1997)

6 $$\begin{aligned} u_{1,m}\left( 0,t\right) =u_{2,m}\left( 0,t\right) =0 \quad \mathrm {for}  \; m\ne 0. \end{aligned}$$

By substituting the series (5) into (4) and applying the pole conditions in (6), we obtain the following ODEs, for each nonnegative integer *m*:

7 $$\begin{aligned} -u_{rr}-\frac{1}{r}u_{r}+\left( \frac{m^{2}}{r^{2}}+\alpha \right) u & = \frac{\partial u}{\partial t}, \quad 0<r<1,\\ u(r,0) & = f(r),\nonumber \\ u\left( 0,t\right) & = 0 \quad \mathrm {if} \;  m\ne 0,\nonumber \\ u\left( 1,t\right) & =  0,\nonumber \end{aligned}$$

where *u* and *f* are now generic functions.

### Weighted formulation

We will extend (7) to the interval $\left( -1,1\right)$ using a coordinate transformation as in Shen (1997). Using the coordinate transformation $r=\frac{s+1}{2}$ in (7) and setting $v(s)=u\left( \frac{s+1}{2}\right)$, we obtain

8 $$\begin{aligned} -v_{ss}-\frac{1}{s+1}v_{s}+\left( \frac{m^{2}}{\left( s+1\right) ^{2}}+\frac{\alpha }{4}\right) v & =  \frac{1}{4}\frac{\partial v}{\partial t}, \quad s\in I=(-1,1),\\ v\left( s,0\right) & = g(s),\nonumber \\ v\left( -1,t\right) & =  0, \quad  \mathrm {if} \quad  m\ne 0,\nonumber \\ v\left( 1,t\right) & = 0,\nonumber \end{aligned}$$

where $g\left( s\right) =f\left( \frac{s+1}{2}\right) .$ To formulate a weighted variational formula for (8), we must find $v\in X\left( m\right)$ such that

9 $$\begin{aligned} \left( \left( s+1\right) v_{s},\left( w\omega \right) _{s}\right) +\left( \frac{m^{2}}{s+1}v,w\right) _{\omega }+\frac{\alpha }{4}\left( \left( s+1\right) v,w\right) _{\omega }=\frac{1}{4}\left( (s+1)\frac{\partial v}{\partial t},w \right) _{\omega } \end{aligned}$$

where $$X\left( m\right) =H_{0,\omega }^{1}\left( I\right) $$ if $$m\ne 0,$$$$X(0)=\left\{ v\in H_{\omega }^{1}\left( I\right) : u\left( 1,t\right) =0\right\} $$ and $$\omega $$ is a weight function.

### Legendre-Galerkin method

To approximate (9) using the Legendre-Galerkin method, we let $\omega =1$ and we have to find $v_{N}\in X_{N}\left( m\right)$ such that $\forall w\in X_{N}\left( m\right)$,

10 $$\begin{aligned} \left( \left( s+1\right) (v_{N})_{s},w_{s}\right) +\left( \frac{m^{2}}{s+1}v_{N},w\right) +&\nonumber \\ \frac{\alpha }{4}\left( \left( s+1\right) v_{N},w\right) _{\omega } & = \left( (s+1)\frac{\partial v_{N}}{\partial t},w\right) , \\ v_{N}\left( s,0\right) & = I_{N}g(s),\nonumber \end{aligned}$$

where $$I_{N}$$ is the interpolation operator based on the Legendre–Gauss–Lobatto points. That is, $$\left( I_{N}g\right) \left( t_{i}\right) =g\left( t_{i}\right) ,$$$$i=0,1,\ldots ,N,$$ where $$\left\{ t_{i}\right\} $$ are the roots of $$\left( 1-t^{2}\right) L_{N}^{\prime }\left( t\right) $$ and $$L_N$$ is the Legendre polynomial of degree *N*.

## The case $m=0$

In the case where $m=0$, (10) reduces to

$$\begin{aligned} \left( \left( s+1\right) \frac{\partial v_{N}}{\partial s},w_{s}\right) +\frac{\alpha }{4}\left( \left( s+1\right) v_{N},w\right) =\left( (s+1)\frac{\partial v_{N}}{\partial t},w\right), \quad \forall w\in X_{N}\left( 0\right) . \end{aligned}$$

As before, we let $L_{k}\left( t\right)$ be the $k\mathrm {th}$-degree Legendre polynomial, and define $X_{N}(0)$ to be the space of all polynomials of degree less than or equal to *N* that vanish at 1. This space can be described as Shen (1997)

$$\begin{aligned} X_{N}\left( 0\right) =\text{ span }\left\{ \phi _{i}\left( t\right) =L_{i}\left( t\right) -L_{i+1}\left( t\right) :\, i=0,1,\ldots ,N-1\right\} , \end{aligned}$$

where $\phi _{i}\left( t\right)$ is the *i*th basis function. By applying the Gram-Schmidt process (Burden, Faires 2005) to these basis functions, $\phi _{i}\left( t\right)$, we can obtain a new set of orthogonal polynomials that will be denoted by $\tilde{\phi }_{i}$, $i=0,1,2,\ldots$, where the degree of $\phi _{i}$ and $\tilde{\phi }_{i}$ is $i+1$. The new basis functions, $\tilde{\phi }_{i}$, can be found by computing

11 $$\begin{aligned} \tilde{\phi }_{i}=\phi _{i}-\sum _{k=0}^{i-1}\frac{\left\langle \tilde{\phi }_{k},\phi _{i}\right\rangle }{\left\langle \tilde{\phi }_{k},\tilde{\phi }_{k}\right\rangle }\tilde{\phi }_{k}. \end{aligned}$$

Fortunately, for $$0\le k\le i-2$$,

12 $$\begin{aligned} \left\langle \tilde{\phi }_{k},\phi _{i}\right\rangle & =  \left\langle \tilde{\phi }_{k},L_{i}-L_{i+1}\right\rangle \nonumber \\ & = \left\langle \tilde{\phi }_{k},L_{i}\right\rangle -\left\langle \tilde{\phi }_{k},L_{i+1}\right\rangle \nonumber \\ & = 0, \end{aligned}$$

due to the orthogonality of the Legendre polynomials, thus greatly simplifying the computation of $\tilde{\phi }_i$.

To start the sequence $\{ \tilde{\phi }_i \}$, we let

$$\begin{aligned} \tilde{\phi }_{0} & =  \phi _{0}\\ & =  L_{0}-L_{1}\\ & =  1-x, \end{aligned}$$

so then

$$\begin{aligned} \tilde{\phi }_{1} & =  \phi _{1}-\frac{\left\langle \tilde{\phi }_{0},\phi _{1}\right\rangle }{\left\langle \tilde{\phi }_{0},\tilde{\phi }_{0}\right\rangle }\tilde{\phi }_{0}\\ & =  \left( -\frac{3}{2}x^{2}+x+\frac{1}{2}\right) -\frac{{-2}/{3}}{{8}/{3}}\left( 1-x\right) \\ & =  -\frac{3}{2}x^{2}+\frac{3}{4}x+\frac{3}{4} \end{aligned}$$

and

$$\begin{aligned} \tilde{\phi }_{2} & =  \phi _{2}-\frac{\left\langle \tilde{\phi }_{0},\phi _{2}\right\rangle }{\left\langle \tilde{\phi }_{0},\tilde{\phi }_{0}\right\rangle }\tilde{\phi }_{0}-\frac{\left\langle \tilde{\phi }_{1},\phi _{2}\right\rangle }{\left\langle \tilde{\phi }_{1},\tilde{\phi }_{1}\right\rangle }\tilde{\phi }_{1}\\ & =  \frac{1}{2}\left( -5x^{3}+3x^{2}+3x-1\right) -\frac{0}{{8}/{3}}\left( 1-x\right) -\frac{-{2}/{5}}{{9}/{10}}\left( -\frac{3}{2}x^{2}+\frac{3}{4}x+\frac{3}{4}\right) \\ & = -\frac{5}{2}x^{3}+\frac{5}{6}x^{2}+\frac{11}{6}x-\frac{1}{6}. \end{aligned}$$

**Fig. 1:**
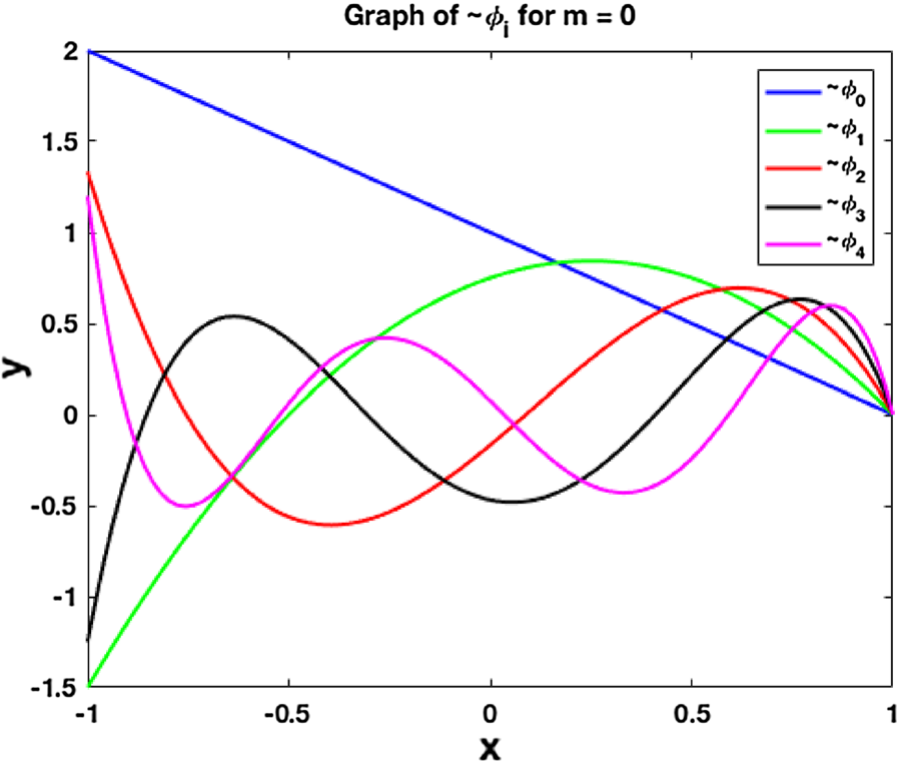
Graphs of $$\tilde{\phi }_i$$, $$i=0,1,2,3,4$$

The first several polynomials $\tilde{\phi }_0, \tilde{\phi }_1,\ldots , \tilde{\phi }_4$ are shown in Fig. 1.

Now, comparing $\phi _{1}$ with $\tilde{\phi }_{1}$ and $\phi _{2}$ with $\tilde{\phi }_{2}$, we can find a general formula for the $\tilde{\phi _{i}}$ in terms of $\phi _i$. By subtracting $\phi _{i}$ from $\tilde{\phi }_{i}$, we obtain

13 $$\begin{aligned} \tilde{\phi }_{1}-\phi _{1} & = -\frac{3}{2}x^{2}+\frac{3}{4}x+\frac{3}{4}-\left( -\frac{3}{2}x^{2}+x+\frac{1}{2}\right) \nonumber \\ & =  -\frac{1}{4}x+\frac{1}{4}\nonumber \\ & =  \frac{1}{4}\tilde{\phi }_{0}, \end{aligned}$$

and

14 $$\begin{aligned} \tilde{\phi }_{2}-\phi _{2} & = -\frac{5}{2}x^{3}+\frac{5}{6}x^{2}+\frac{11}{6}x-\frac{1}{6}-\left( -\frac{5}{2}x^{3}+\frac{3}{2}x^{2}+\frac{3}{2}x-\frac{1}{2}\right) \nonumber \\ & =  \frac{4}{9}\left( -\frac{3}{2}x^{2}+\frac{3}{4}x+\frac{3}{4}\right) \nonumber \\ & =  \frac{4}{9}\tilde{\phi }_{1}. \end{aligned}$$

This suggests a simple recurrence relation for $\tilde{\phi }_i$ in terms of $\phi _i$. Before we prove that this relation holds in general, we need the following result.

### **Lemma 1**

*Let*$$N_{k}=\left\langle \tilde{\phi }_{k},\tilde{\phi }_{k}\right\rangle $$. *Then*

15 $$\begin{aligned} N_{k}=\frac{2\left( k+2\right) ^{2}}{\left( k+1\right) ^{2}\left( 2k+3\right) }, \end{aligned}$$

$$\forall k\ge 0.$$

### *Proof*

We proceed by induction. For the base case, we have

$$\begin{aligned} N_{0} & =  \left\langle \tilde{\phi }_{0},\tilde{\phi }_{0},\right\rangle \\ & =  \int _{-1}^{1}\tilde{\phi }_{0}(x) \tilde{\phi }_{0}(x)\,dx\\ & =  \int _{-1}^{1}\left( 1-x\right) \left( 1-x\right) \,dx\\ & =  \frac{8}{3}. \end{aligned}$$

For the induction step, we assume that there is a $k > 0,$ such that $N_{k-1}=\frac{2\left( k+1\right) ^{2}}{k^{2}\left( 2k+1\right) }$. We must show that the formula found in Eq. (15) is true for *k*. Given $\tilde{\phi }_{k}=\phi _{k}+\left( \frac{k}{k+1}\right) ^{2}\tilde{\phi }_{k-1},$ and using

16 $$\begin{aligned} \langle L_k, L_k \rangle = \frac{2}{2k+1}, \end{aligned}$$

we have

$$\begin{aligned} N_{k} & =  \left\langle \tilde{\phi }_{k},\tilde{\phi }_{k}\right\rangle \\ & =  \left\langle \phi _{k}+\left( \frac{k}{k+1}\right) ^{2}\tilde{\phi }_{k-1},\phi _{k}+\left( \frac{k}{k+1}\right) ^{2}\tilde{\phi }_{k-1}\right\rangle \\ & =  \left\langle \phi _{k},\phi _{k}\right\rangle +2\left( \frac{k}{k+1}\right) ^{2}\left\langle \phi _{k},\tilde{\phi }_{k-1}\right\rangle +\left( \frac{k}{k+1}\right) ^{4}\left\langle \tilde{\phi }_{k-1},\tilde{\phi }_{k-1}\right\rangle \\ & =  \left\langle L_{k}+L_{k+1},L_{k}+L_{k+1}\right\rangle -2\left( \frac{k}{k+1}\right) ^{2}\left\langle L_{k},L_{k}\right\rangle +\left( \frac{k}{k+1}\right) ^{4}N_{k-1}\\ & =  \frac{8\left( k+1\right) }{\left( 2k+1\right) \left( 2k+3\right) }-\frac{4k^{2}}{\left( k+1\right) ^{2}\left( 2k+1\right) }+\left( \frac{k}{k+1}\right) ^{4}N_{k-1}\\ & =  \frac{4\left( 3k^{2}+6k+2\right) }{\left( k+1\right) ^{2}\left( 2k+1)\right) \left( 2k+3\right) }+\left( \frac{k}{k+1}\right) ^{4}N_{k-1}\\ & =  \frac{2\left( k+2\right) ^{2}}{\left( k+1\right) ^{2}\left( 2k+3\right) }. \end{aligned}$$

$$\square $$

We can now establish the pattern seen in (13), (14).

### **Theorem 1**

*If*$\tilde{\phi }_0(x) = 1-x$*and*$\tilde{\phi }_i$*is obtained by orthogonalizing*$\phi _i = L_{i+1} - L_i$*against*$\tilde{\phi }_0, \tilde{\phi }_0, \ldots , \tilde{\phi }_{i-1}$, *then*

17 $$\begin{aligned} \tilde{\phi }_{i}=\phi _{i}+c_{i}\tilde{\phi }_{i-1} \end{aligned}$$

*for*$$i=1,2,\ldots ,$$*where*$$c_{i}=\left( \frac{i}{i+1}\right) ^{2}$$.

### *Proof*

Again we proceed by induction. For the base case, we will show that the theorem holds when $i=1$:

18 $$\begin{aligned} \tilde{\phi }_{1} & =  \phi _{1}-\frac{\left\langle \tilde{\phi }_{0},\phi _{1}\right\rangle }{\left\langle \tilde{\phi }_{0},\tilde{\phi }_{0}\right\rangle }\tilde{\phi }_{0}\nonumber \\ & =  x-\frac{3}{2}x^{2}+\frac{1}{2}-\frac{{-2}/{3}}{{8}/{3}}\left( 1-x\right) \nonumber \\ & =  x-\frac{3}{2}x^{2}+\frac{1}{2}+\frac{1}{4}\left( 1-x\right) \nonumber \\ & =  \phi _{1}+\frac{1}{4}\tilde{\phi }_{0}. \end{aligned}$$

Note that Eq. (18) is equivalent to Eq. (13). For the induction step, we assume that there is a $j \ge 0$, such that

19 $$\begin{aligned} \tilde{\phi }_{j}=\phi _{j}+\left( \frac{j}{j+1}\right) ^{2}\tilde{\phi }_{j-1}. \end{aligned}$$

We show that (17) holds when $i=j+1.$ We have

$$\begin{aligned} \tilde{\phi }_{j+1} & =  \phi _{j+1}+\sum _{k=0}^{j}\frac{\left\langle \phi _{j+1},\tilde{\phi }_{k}\right\rangle }{\left\langle \tilde{\phi }_{k},\tilde{\phi }_{k}\right\rangle }\tilde{\phi }_{k}\\ & =  \phi _{j+1}-\frac{\left\langle \phi _{j+1},\tilde{\phi }_{j}\right\rangle }{\left\langle \tilde{\phi }_{j},\tilde{\phi }_{j}\right\rangle }\tilde{\phi }_{j}\\ & =  \phi _{j+1}-\frac{\left\langle L_{j+1},\tilde{\phi }_{j}\right\rangle -\left[ \left\langle L_{j+2},\phi _{j}\right\rangle +(\frac{j}{j+1})^{2}\left\langle L_{j+2},\tilde{\phi }_{j-1}\right\rangle \right] }{\left\langle \tilde{\phi }_{j},\tilde{\phi }_{j}\right\rangle }\tilde{\phi }_{j}\\ & =  \phi _{j+1}-\frac{\left\langle L_{j+1},\tilde{\phi }_{j}\right\rangle }{\left\langle \tilde{\phi }_{j},\tilde{\phi }_{j}\right\rangle }\tilde{\phi }_{j}\\ & =  \phi _{j+1}-\frac{\left\langle L _{j+1},\phi _{j} \right\rangle +(\frac{j}{j+1})^2\left\langle L_{j+1},\tilde{\phi }_{j-1}\right\rangle }{\left\langle \tilde{\phi }_{j},\tilde{\phi }_{j}\right\rangle }\tilde{\phi }_{j}\\ & =  \phi _{j+1}-\frac{\left\langle L _{j+1},\phi _{j} \right\rangle }{\left\langle \tilde{\phi }_{j},\tilde{\phi }_{j}\right\rangle }\tilde{\phi }_{j}\\ & =  \phi _{j+1}-\frac{\left\langle L _{j+1},L_{j}\right\rangle -\left\langle L_{j+1},L_{j+1}\right\rangle }{\left\langle \tilde{\phi }_{j},\tilde{\phi }_{j}\right\rangle }\tilde{\phi }_{j}\\ & =  \phi _{j+1}+\frac{\left\langle L_{j+1},L_{j+1}\right\rangle }{\left\langle \tilde{\phi }_{j},\tilde{\phi }_{j}\right\rangle }\tilde{\phi }_{j}.\end{aligned}$$

Therefore, using Lemma 1 and (16), we obtain

$$\begin{aligned} \tilde{\phi }_{j+1} & = \phi _{j+1}+\frac{\left( \frac{2}{2\left( j+1\right) +1}\right) }{\left\langle \tilde{\phi }_{j},\tilde{\phi }_{j}\right\rangle }\tilde{\phi }_{j}\\ & =  {} \phi _{j+1}+\frac{2}{\left( 2j+3\right) }\frac{\left( j+1\right) ^{2}\left( 2j+3\right) }{2\left( j+2\right) ^{2}}\tilde{\phi }_{k}\\ & =  {} \phi _{j+1}+\left( \frac{j+1}{j+2}\right) ^{2}\tilde{\phi }_{j}. \, \end{aligned}$$

$$\square $$

We now prove a converse of Theorem 1.

### **Theorem 2**

*If*$\tilde{\phi }_0(x) = 1-x$*and*$\tilde{\phi }_i$*is defined as in* (17) *for*$i=1,2,\ldots ,$*then*$\left\langle \tilde{\phi }_{k},\tilde{\phi }_{j}\right\rangle =0$*when*$j<k.$

### *Proof*

Case 1: $$j<k-1$$

$$\begin{aligned} \left\langle \tilde{\phi }_{k},\tilde{\phi }_{j}\right\rangle & =  {} \left\langle \phi _{k}+\left( \frac{k}{k+1}\right) ^{2}\tilde{\phi }_{k-1},\phi _{j}+\left( \frac{j}{j+1}\right) ^{2}\tilde{\phi }_{j-1}\right\rangle \\ & =  {} \left\langle \phi _{k},\phi _{j}\right\rangle +\left( \frac{k}{k+1}\right) ^{2}\left\langle \tilde{\phi }_{k-1},\phi _{j}\right\rangle \\ & =  {} \left\langle L_{k},L_{j}\right\rangle -\left\langle L_{k},L_{j+1}\right\rangle -\left\langle L_{k+1},L_{j}\right\rangle +\left\langle L_{k+1},L_{j+1}\right\rangle +\left( \frac{k}{k+1}\right) ^{2}\left\langle \tilde{\phi }_{k-1},\phi _{j}\right\rangle \\ & =  {} \left( \frac{k}{k+1}\right) ^{2}\left\langle \tilde{\phi }_{k-1},\phi _{j}\right\rangle \\ & =  {} 0. \end{aligned}$$

Case 2: $$j=k-1$$

$$\begin{aligned} \left\langle \tilde{\phi }_{k},\tilde{\phi }_{j}\right\rangle & =  {} \left\langle \phi _{k},\phi _{k-1}\right\rangle +\left( \frac{k}{k+1}\right) ^{2}\left\langle \tilde{\phi }_{k-1},\phi _{k-1}\right\rangle \\ & =  {} \left\langle L_{k}-L_{k+1},L_{k-1}-L_{k}\right\rangle +\left( \frac{k}{k+1}\right) ^{2}\left\langle \tilde{\phi }_{k-1},\phi _{k-1}\right\rangle \\ & =  {} -\left\langle L_{k},L_{k}\right\rangle +\left( \frac{k}{k+1}\right) ^{2}\left\langle \tilde{\phi }_{k-1},\tilde{\phi }_{k-1}\right\rangle \\ & =  {} -\left( \frac{2}{2k+1}\right) +\left( \frac{k}{k+1}\right) ^{2}\left( \frac{2\left( \left( k-1\right) +2\right) ^{2}}{\left( \left( k-1\right) +1\right) ^{2}\left( 2\left( k-1\right) +3\right) }\right) \\ & =  {} 0. \end{aligned}$$

$$\square $$

All orthogonal polynomials satisfy a general three-term recurrence relation that has the form

20 $$\begin{aligned} \beta _{j}\tilde{\phi }_{j+1}(x)=\left( x-\alpha _{j}\right) \tilde{\phi }_{j}(x)-\gamma _{j-1}\tilde{\phi }_{j-1}(x), \end{aligned}$$

where $\alpha _{j}$, $\beta _{j}$ and $\gamma _j$ are constants. By enforcing orthogonality, we obtain the formulas

21 $$\begin{aligned} \alpha _j & =  {} \frac{\langle \tilde{\phi }_j, x\tilde{\phi }_j \rangle }{\langle \tilde{\phi }_j, \tilde{\phi }_j \rangle }, \end{aligned}$$

22 $$\begin{aligned} \beta _j & =  {} \frac{\langle \tilde{\phi }_{j+1}, x\tilde{\phi }_j \rangle }{\langle \tilde{\phi }_{j+1}, \tilde{\phi }_{j+1} \rangle }, \end{aligned}$$

23 $$\begin{aligned} \gamma _j & =  {} \frac{\langle \tilde{\phi }_{j+1}, x\tilde{\phi }_j \rangle }{\langle \tilde{\phi }_j, \tilde{\phi }_j \rangle }, . \end{aligned}$$

First, we will find the value of $\alpha _{j}$.

### **Theorem 3**

*Let*$\alpha _j$*be defined as in* (21). *Then*$\alpha _{j}=-\frac{1}{\left( j+1\right) \left( j+2\right) },\; \forall j\ge 0.$

### *Proof*

Base case: When $j=0$, we use (21) to obtain

$$\begin{aligned} \alpha _{0} & =  {} \frac{\left\langle \tilde{\phi }_{0},x\tilde{\phi }_{0}\right\rangle }{\left\langle \tilde{\phi }_{0},\tilde{\phi }_{0}\right\rangle }\\ & =  {} \frac{\int _{-1}^{1}\tilde{\phi }_{0}(x) x\tilde{\phi }_{0}(x)\,dx}{\int _{-1}^{1}\tilde{\phi }_{0}(x) \tilde{\phi }_{0}(x)\,dx}\\ & =  {} \frac{\int _{-1}^{1}\left( x^{3}-2x^{2}+x\right) \,dx}{\int _{-1}^{1}\left( x^{2}-2x+1\right) \,dx}\\ & =  {} -\frac{1}{2}. \end{aligned}$$

For the induction hypothesis, we assume there is a $j > 0$ such that $\alpha _{j-1}=-\frac{1}{j\left( j+1\right) }$. From $\alpha _{j}=\frac{\left\langle \tilde{\phi }_{j},x\tilde{\phi }_{j}\right\rangle }{\left\langle \tilde{\phi }_{j},\tilde{\phi }_{j}\right\rangle }$ and $\tilde{\phi }_{j}=\phi _{j}+c_{j}\tilde{\phi }_{j-1}$, where $c_{j}=\left( \frac{j}{j+1}\right) ^{2}$, we obtain

24 $$\begin{aligned} \left\langle \tilde{\phi }_{j},x\tilde{\phi }_{j}\right\rangle & =  {} \left\langle \phi _{j}+c_{j}\tilde{\phi }_{j-1},x\left( \phi _{j}+c_{j}\tilde{\phi }_{j-1}\right) \right\rangle \nonumber \\ & =  {} \left\langle \phi _{j}+c_{j}\tilde{\phi }_{j-1},x\phi _{j}+xc_{j}\tilde{\phi }_{j-1}\right\rangle \nonumber \\ & =  {} \left\langle \phi _{j},x\phi _{j}\right\rangle +2c_{j}\left\langle \tilde{\phi }_{j-1},x\phi _{j}\right\rangle +c_{j}^{2}\left\langle \tilde{\phi }_{j-1},x\tilde{\phi }_{j-1}\right\rangle . \end{aligned}$$

Now, from the recurrence relation for Legendre polynomials, we obtain

25 $$\begin{aligned} \left\langle \phi _{j},x\phi _{j}\right\rangle & =  {} \left\langle L_{j}-L_{j+1},x\left( L_{j}-L_{j+1}\right) \right\rangle \nonumber \\ & =  {} \left\langle L_{j}-L_{j+1},\left( \frac{j+1}{2j+1}L_{j+1}+\frac{j}{2j+1}L_{j-1}\right) -\left( \frac{j+2}{2j+3}L_{j+2}+\frac{j+1}{2j+3}L_{j}\right) \right\rangle \nonumber \\ & =  {} -\left( \frac{j+1}{2j+3}\right) \left\langle L_{j},L_{j}\right\rangle -\left( \frac{j+1}{2j+1}\right) \left\langle L_{j+1},L_{j+1}\right\rangle \nonumber \\ & =  {} -\left( \frac{j+1}{2j+3}\right) \left( \frac{2}{2j+1}\right) -\left( \frac{j+1}{2j+1}\right) \left( \frac{2}{2j+3}\right) \nonumber \\ & =  {} \frac{-4(j+1)}{(2j+1)(2j+3)}. \end{aligned}$$

and

26 $$\begin{aligned} \left\langle \tilde{\phi }_{j-1},x\phi _{j}\right\rangle & =  {} \left\langle \tilde{\phi }_{j-1},\left( \frac{j+1}{2j+1}L_{j+1}+\frac{j}{2j+1}L_{j-1}\right) -\left( \frac{j+2}{2j+3}L_{j+2}+\frac{j+1}{2j+3}L_{j}\right) \right\rangle \nonumber \\ & =  {} \frac{j}{2j+1}\left\langle \tilde{\phi }_{j-1},L_{j-1}\right\rangle -\frac{j+1}{2j+3}\left\langle \tilde{\phi }_{j-1},L_{j}\right\rangle \nonumber \\ & =  {} \frac{j}{2j+1}\left\langle \tilde{\phi }_{j-1},L_{j-1}\right\rangle -\frac{j+1}{2j+3}\left\langle \phi _{j-1}+\left( \frac{j-1}{j}\right) ^{2}\tilde{\phi }_{j-2},L_{j}\right\rangle \nonumber \\ & =  {} \frac{j}{2j+1}\left\langle \tilde{\phi }_{j-1},L_{j-1}\right\rangle -\frac{j+1}{2j+3}\left[ \left\langle \phi _{j-1},L_{j}\right\rangle \ +\left( \frac{j-1}{j}\right) ^{2}\left\langle \tilde{\phi }_{j-2},L_{j}\right\rangle \right] \nonumber \\ & =  {} \frac{j}{2j+1}\left\langle \tilde{\phi }_{j-1},L_{j-1}\right\rangle -\frac{j+1}{2j+3}\left\langle \phi _{j-1},L_{j}\right\rangle \nonumber \\ & =  {} \frac{j}{2j+1}\left\langle \tilde{\phi }_{j-1},L_{j-1}\right\rangle +\frac{j+1}{2j+3}\left\langle L_{j},L_{j}\right\rangle \nonumber \\ & =  {} \frac{j}{2j+1}\left[ \left\langle L_{j-1},L_{j-1}\right\rangle -c_{j-1}\left\langle L_{j-1},L_{j-1}\right\rangle \right] +\frac{j+1}{2j+3}\left\langle L_{j},L_{j}\right\rangle \nonumber \\ & =  {} \frac{j}{2j+1}\left[ \left( 1-c_{j-1}\right) \left\langle L_{j-1},L_{j-1}\right\rangle \right] +\frac{j+1}{2j+3}\left\langle L_{j},L_{j}\right\rangle \nonumber \\ & =  {} \frac{j}{2j+1}\left[ \left( 1-\left( \frac{j-1}{j}\right) ^{2}\right) \left( \frac{2}{2j-1}\right) \right] +\frac{j+1}{2j+3}\left( \frac{2}{2j+1}\right) \nonumber \\ & =  {} \frac{2(j^{2}+3j+3)}{j\left( 2j+1\right) \left( 2j+3\right) }. \end{aligned}$$

To calculate the middle term in Eq. (24) we will multiply $2c_{j}$ by the result from Eq. (26):

27 $$\begin{aligned} 2c_{j}\left\langle \tilde{\phi }_{j-1},x\phi _{j}\right\rangle & =  {} 2\left( \frac{j}{j+1}\right) ^{2}\left( \frac{2\left( j^{2}+3j+3\right) }{j\left( 2j+1\right) \left( 2j+3\right) }\right) \nonumber \\ & =  {} \frac{4j\left( j^{2}+3j+3\right) }{\left( j+1\right) ^{2}\left( 2j+1\right) \left( 2j+3\right) }. \end{aligned}$$

We rearrange the formula for $\alpha _{j-1}$ to obtain the following:

$$\begin{aligned} \left\langle \tilde{\phi }_{j-1},x\tilde{\phi }_{j-1}\right\rangle & =  {} \alpha _{j-1}\left\langle \tilde{\phi }_{j-1},\tilde{\phi }_{j-1}\right\rangle \\ & =  {} -\frac{1}{j\left( j+1\right) }\frac{2(\left( j+1\right) ^{2}}{j^{2}\left( 2j+1\right) }\\ & =  {} \frac{-2\left( j+1\right) }{j^{3}\left( 2j+1\right) }. \end{aligned}$$

Therefore,

28 $$\begin{aligned} c_{j}^{2}\left\langle \tilde{\phi }_{j-1},x\tilde{\phi }_{j-1}\right\rangle & =  {} \left( \frac{j}{j+1}\right) ^{4}\left( \frac{-2\left( j+1\right) }{j^{3}\left( 2j+1\right) }\right) \nonumber \\ & =  {} \frac{-2j}{\left( j+1\right) ^{3}\left( 2j+1\right) }. \end{aligned}$$

Now we can use the results from Eqs. (25)–(28) to determine the numerator of $\alpha _{j}.$

$$\begin{aligned} \left\langle \tilde{\phi }_{j},x\tilde{\phi }_{j}\right\rangle & =   \frac{-4(j+1)}{(2j+1)(2j+3)}+\frac{4j\left( j^{2}+3j+3\right) }{\left( j+1\right) ^{2}\left( 2j+1\right) \left( 2j+3\right) }+\frac{-2j}{\left( j+1\right) ^{3}\left( 2j+1\right) }\\ & =  {} \frac{-2\left( j+2\right) }{\left( j+1\right) ^{3}\left( 2j+3\right) }. \end{aligned}$$

Hence,

$$\begin{aligned} \alpha _{j} & =  {} \frac{-2\left( j+2\right) }{\left( j+1\right) ^{3}\left( 2j+3\right) }\frac{\left( j+1\right) ^{2}\left( 2j+3\right) }{2\left( j+2\right) ^{2}}\\ & =  {} -\frac{1}{\left( j+1\right) \left( j+2\right) }. \, \end{aligned}$$

$$\square $$

Now, we will find the value of $\beta _{k}$.

### **Theorem 4**

*Let*$\beta _j$*be defined as in* (22). *Then*$\beta _{j}=\frac{j+2}{2j+3},\; \forall j\ge 0.$

### *Proof*

For the base case, we consider $j=0$:

$$\begin{aligned} \beta _{0} & =  {} \frac{\left\langle \tilde{\phi }_{1},x\tilde{\phi }_{0}\right\rangle }{\left\langle \tilde{\phi }_{1},\tilde{\phi }_{1}\right\rangle }\\ & =  {} \frac{\int _{-1}^{1}\tilde{\phi }_{1}(x) x\tilde{\phi }_{0}(x)\,dx}{\int _{-1}^{1}\tilde{\phi }_{1}(x) \tilde{\phi }_{1}(x)\,dx}\\ & =  {} \frac{\int _{-1}^{1}\left( -\frac{3}{2}x^{2}+\frac{3}{4}x+\frac{3}{4}\right) x\left( 1-x\right) \,dx}{\int _{-1}^{1}\left( -\frac{3}{2}x^{2}+\frac{3}{4}x+\frac{3}{4}\right) \left( -\frac{3}{2}x^{2}+\frac{3}{4}x+\frac{3}{4}\right) \,dx}\\ & =  {} \frac{2}{3}. \end{aligned}$$

For the induction step, we assume there is a $j\ge 0$ such that $\beta _{j-1}=\frac{j+1}{2j+1}$. From $\beta _{j}=\frac{\left\langle \tilde{\phi }_{j+1},x\tilde{\phi }_{j}\right\rangle }{\left\langle \tilde{\phi }_{j+1},\tilde{\phi }_{j+1}\right\rangle }$ and $\tilde{\phi }_{j}=\phi _{j}+c_{j}\tilde{\phi }_{j-1}$ where $c_{j}=\left( \frac{j}{j+1}\right) ^{2},$ we obtain

29 $$\begin{aligned} \left\langle \tilde{\phi }_{j+1},x\tilde{\phi }_{j}\right\rangle & =  {} \left\langle \phi _{j+1}+c_{j+1}\tilde{\phi }_{j},x\left( \phi _{j}+c_{j}\tilde{\phi }_{j-1}\right) \right\rangle \nonumber \\ & =  {} \left\langle \phi _{j+1},x\phi _{j}\right\rangle +c_{j}\left\langle \tilde{\phi }_{j-1},x\phi _{j+1}\right\rangle +c_{j+1}\left\langle \tilde{\phi }_{j},x\phi _{j}\right\rangle \nonumber \\& \quad +c_{j}c_{j+1}\left\langle \tilde{\phi }_{j},x\tilde{\phi }_{j-1}\right\rangle . \end{aligned}$$

Using the recurrence relation for Legendre polynomials, we obtain

30 $$\begin{aligned} \left\langle \phi _{j+1},x\phi _{j}\right\rangle & =  {} \left\langle L_{j+1}-L_{j+2},x\left( L_{j}-L_{j+1}\right) \right\rangle \nonumber \\ & =  {} \left\langle L_{j+1}-L_{j+2},\left( \frac{j+1}{2j+1}L_{j+1}+\frac{j}{2j+1}L_{j-1}\right) -\left( \frac{j+2}{2j+3}L_{j+2}+\frac{j+1}{2j+3}L_{j}\right) \right\rangle \nonumber \\ & =  {} \frac{j+1}{2j+1}\left\langle L_{j+1},L_{j+1}\right\rangle +\frac{j+2}{2j+3}\left\langle L_{j+2},L_{j+2}\right\rangle \nonumber \\ & =  {} \frac{j+1}{2j+1}\left( \frac{2}{2j+3}\right) +\frac{j+2}{2j+3}\left( \frac{2}{2j+5}\right) \nonumber \\ & =  {} \frac{2(4j^{2}+12j+7)}{(2j+1)(2j+3)\left( 2j+5\right) }.\end{aligned}$$

and

$$\begin{aligned} \left\langle \tilde{\phi }_{j-1},x\phi _{j+1}\right\rangle & =  {} \left\langle \tilde{\phi }_{j-1},xL_{j+1}-xL_{j+2}\right\rangle \\ & =  {} \left\langle \tilde{\phi }_{j-1},\left( \frac{j+2}{2j+3}L_{j+2}+\frac{j+1}{2j+3}L_{j}\right) -\left( \frac{j+3}{2j+5}L_{j+3}+\frac{j+2}{2j+5}L_{j+1}\right) \right\rangle \\ & =  {} \frac{j+1}{2j+3}\left\langle \tilde{\phi }_{j-1},L_{j}\right\rangle \\ & =  {} \frac{j+1}{2j+3}\left\langle \phi _{j-1}+\left( \frac{j-1}{j}\right) ^{2}\tilde{\phi }_{j-2},L_{j}\right\rangle \\ & =  {} \frac{j+1}{2j+3}\left\langle \phi _{j-1},L_{j}\right\rangle \\ & =  {} -\frac{j+1}{2j+3}\left\langle L_{j},L_{j}\right\rangle \\ & =  {} -\frac{j+1}{2j+3}\left( \frac{2}{2j+1}\right) \\ & =  {} -\frac{2\left( j+1\right) }{\left( 2j+1\right) \left( 2j+3\right) }. \end{aligned}$$

We then have

31 $$\begin{aligned} c_{j}\left\langle \tilde{\phi }_{j-1},x\phi _{j+1}\right\rangle & =  {} \left( \frac{j}{j+1}\right) ^{2}\left( -\frac{2\left( j+1\right) }{\left( 2j+1\right) \left( 2j+3\right) }\right) \nonumber \\ & =  {} \frac{-2j^{2}}{\left( j+1\right) \left( 2j+1\right) \left( 2j+3\right) }.\end{aligned}$$

The last term in (29) is obtained as follows:

$$\begin{aligned} \left\langle \tilde{\phi }_{j},x\phi _{j}\right\rangle & =  {} \left\langle \tilde{\phi }_{j},\left( \frac{j+1}{2j+1}L_{j+1}+\frac{j}{2j+1}L_{j-1}\right) -\left( \frac{j+2}{2j+3}L_{j+2}+\frac{j+1}{2j+3}L_{j}\right) \right\rangle \\ & =  {} \frac{j+1}{2j+1}\left\langle \tilde{\phi }_{j},L_{j+1}\right\rangle +\frac{j}{2j+1}\left\langle \tilde{\phi }_{j},L_{j-1}\right\rangle -\frac{j+1}{2j+3}\left\langle \tilde{\phi }_{j},L_{j}\right\rangle \\ & =  {} \frac{j+1}{2j+1}\left( -\left\langle L_{j+1},L_{j+1}\right\rangle \right) +\frac{j}{2j+1}\left[ c_{j}\left( 1-c_{j-1}\right) \left\langle L_{j-1},L_{j-1}\right\rangle \right] -\frac{j+1}{2j+3}\left[ \left( 1-c_{j}\right) \left\langle L_{j},L_{j}\right\rangle \right] \\ & =  {} -\frac{j+1}{2j+1}\left( \frac{2}{2j+3}\right) +\frac{j}{2j+1}\left( \frac{2}{\left( j+1\right) ^{2}}\right) -\frac{j+1}{2j+3}\left( \frac{2}{\left( j+1\right) ^{2}}\right) \\ & =  {} -\frac{2\left( j+2\right) \left( j^{2}+j+1\right) }{\left( j+1\right) ^{2}\left( 2j+1\right) \left( 2j+3\right) }. \end{aligned}$$

We then have

32 $$\begin{aligned} c_{j+1}\left\langle \tilde{\phi }_{j},x\phi _{j}\right\rangle & =  {} \left( \frac{j+1}{j+2}\right) ^{2}\left( -\frac{2\left( j+2\right) \left( j^{2}+j+1\right) }{\left( j+1\right) ^{2}\left( 2j+1\right) \left( 2j+3\right) }\right) \nonumber \\ & =  {} \frac{-2\left( j^{2}+j+1\right) }{\left( j+2\right) \left( 2j+1\right) \left( 2j+3\right) }. \end{aligned}$$

We rearrange the formula for $\beta _{j-1}$ to obtain the following:

$$\begin{aligned} \left\langle \tilde{\phi }_{j},x\tilde{\phi }_{j-1}\right\rangle & =  {} \beta _{j-1}\left\langle \tilde{\phi }_{j},\tilde{\phi }_{j}\right\rangle \\ & =  {} \frac{j+1}{2j+1}\frac{2(\left( j+2\right) ^{2}}{\left( j+1\right) ^{2}\left( 2j+3\right) }\\ & =  {} \frac{2\left( j+2\right) ^{2}}{\left( j+1\right) \left( 2j+1\right) \left( 2j+3\right) }. \end{aligned}$$

Therefore,

33 $$\begin{aligned} c_{j}c_{j+1}\left\langle \tilde{\phi }_{j},x\tilde{\phi }_{j-1}\right\rangle & =  {} \left( \frac{j}{j+1}\right) ^{2}\left( \frac{j+1}{j+2}\right) ^{2}\left( \frac{2\left( j+2\right) ^{2}}{\left( j+1\right) \left( 2j+1\right) \left( 2j+3\right) }\right) \nonumber \\ & =  {} \frac{2j^{2}}{\left( j+1\right) \left( 2j+1\right) \left( 2j+3\right) }. \end{aligned}$$

Now we can use the results from Eqs. (30)–(33) to determine the numerator of $\beta _{j}.$

$$\begin{aligned} \left\langle \tilde{\phi }_{j+1},x\tilde{\phi }_{j}\right\rangle & =  {} \frac{2(4j^{2}+12j+7)}{(2j+1)(2j+3)\left( 2j+5\right) }-\frac{2j^{2}}{\left( j+1\right) \left( 2j+1\right) \left( 2j+3\right) }\\&-\frac{2\left( j^{2}+j+1\right) }{\left( j+2\right) \left( 2j+1\right) \left( 2j+3\right) } +\frac{2j^{2}}{\left( j+1\right) \left( 2j+1\right) \left( 2j+3\right) }\\ & =  {} \frac{2\left( j+3\right) ^{2}}{\left( j+2\right) \left( 2j+3\right) \left( 2j+5\right) } \end{aligned}$$

Hence,

$$\begin{aligned} \beta _{j} & =  {} \frac{2\left( j+3\right) ^{2}}{\left( j+2\right) \left( 2j+3\right) \left( 2j+5\right) }\frac{\left( j+2\right) ^{2}\left( 2j+5\right) }{2\left( j+3\right) ^{2}}\\ & =  {} \frac{j+2}{2j+3}.\, \end{aligned}$$

$$\square $$

Using the same approach as in the preceding proof, we obtain

34 $$\begin{aligned} \gamma _j & =  {} \frac{2\left( j+3\right) ^{2}}{\left( j+2\right) \left( 2j+3\right) \left( 2j+5\right) } \frac{\left( j+1\right) ^{2}\left( 2j+3\right) }{2\left( j+2\right) ^{2}} \nonumber \\ & =  {} \frac{\left( j+1\right) ^{2}(j+3)^2}{\left( j+2\right) ^3\left( 2j+5\right) }. \end{aligned}$$

In summary, the polynomials $\{ \tilde{\phi }_i \}$ satisfy the recurrence relation

35 $$\begin{aligned} \frac{j+2}{j+3}\tilde{\phi }_{j+1}(x) = \left( x + \frac{1}{(j+1)(j+2)} \right) \tilde{\phi }_j(x) - \frac{j^{2}(j+2)^2}{\left( j+1\right) ^3\left( 2j+3\right) } \tilde{\phi }_{j-1}(x). \end{aligned}$$

We can rewrite Eq. (19) as $\tilde{\phi }_{j}-c_{j}\tilde{\phi }_{j-1}=\phi _{j}$. In matrix form, we have

36 $$\begin{aligned} \varPhi =\tilde{\varPhi }C, \quad C = \left[ \begin{array}{ccccc} 1 &{} -c_{1}\\ &{} 1 &{} -c_{2}\\ &{} &{} 1 &{} \ddots \\ &{} &{} &{} \ddots &{} -c_{n}\\ &{} &{} &{} &{} 1\end{array}\right] , \end{aligned}$$

where $$ \varPhi =\left[ \begin{array}{cccc} \phi _{0}(\mathbf {x})&\varphi _{1}(\mathbf {x})&\cdots&\phi _{n}(\mathbf {x}) \end{array}\right] $$ and $$\tilde{\varPhi }=\left[ \begin{array}{cccc}\tilde{\phi }_{0}(\mathbf {x})&\tilde{\varphi }_{1}(\mathbf {x})&\cdots&\tilde{\varphi }_{n}(\mathbf {x})\end{array}\right] $$, with $\mathbf{x}$ being a vector of at least $n+2$ Legendre–Gauss–Lobatto points. This ensures that the columns of $\tilde{\varPhi }$ are orthogonal.

Then, given $f \in X_{n+1}(0)$, we can obtain the coefficients $\tilde{f}_i$ in

$$\begin{aligned} f(x) = \sum _{i=0}^n \tilde{f}_i \tilde{\phi }_i(x) \end{aligned}$$

by simply computing $\tilde{f}_i = \langle \tilde{\phi }_i, f \rangle / N_i$, where $N_i$ is as defined in (15). Then the coefficients $f_i$ in

$$\begin{aligned} f(x) = \sum _{i=0}^n f_i \phi _i(x) \end{aligned}$$

can be obtained by solving the system $C\mathbf{f} = \tilde{\mathbf{f}}$ using back substitution, where *C* is as defined in (36). These coefficients can be used in conjunction with the discretization used in Shen (1997), which makes use of the basis $\{ \phi _i \}$.

## The case $m\ne 0$

In the case where $m\ne 0$, we work with the space

$$\begin{aligned} X_N(m) = \{ p\in \mathcal{P}_N | p(-1) = p(1) = 0 \}. \end{aligned}$$

As discussed in Shen (1997), this space can easily be described in terms of Legendre polynomials:

$$\begin{aligned} X_{N}\left( m\right) =\mathrm {span}\left\{ \phi _{i}\left( t\right) =L_{i}\left( t\right) -L_{i+2}\left( t\right) , \quad  i=0,1,\ldots ,N-2\right\} \end{aligned}$$

Applying the Gram-Schmidt process to the basis functions $\{ \phi _i \}$, we obtain a new set of orthogonal polynomials that will be denoted as $\{ \hat{\phi }_{i} \}$. These basis functions are obtained in the same way as in Eq. (11). First, we let

$$\begin{aligned} \tilde{\phi }_{0} & =  {} \phi _{0}\\ & =  {} L_{0}-L_{2}\\ & =  {} -\frac{3}{2}x^{2}+\frac{3}{2} \end{aligned}$$

and

$$\begin{aligned} \hat{\phi }_{1} & =  {} \phi _{1}\\ & =  {} L_{1}-L_{3}\\ & =  {} -\frac{5}{2}x^{3}+\frac{5}{2}x. \end{aligned}$$

Then, we have

$$\begin{aligned} \hat{\phi }_{2} & =  {} \phi _{2}-\frac{\left\langle \hat{\phi }_{0},\phi _{2}\right\rangle }{\left\langle \hat{\phi }_{0},\hat{\phi }_{0}\right\rangle }\hat{\phi }_{0}-\frac{\left\langle \hat{\phi }_{1},\phi _{2}\right\rangle }{\left\langle \hat{\phi }_{1},\hat{\phi }_{1}\right\rangle }\hat{\phi }_{1}\\ & =  {} \phi _{2}-\frac{\int _{1}^{-1}\left( \hat{\phi }_{0}(x)\phi _{2}(x)\right) \,dx}{\int _{1}^{-1}\left( \hat{\phi }_{0}(x)\hat{\phi }_{0}(x)\right) \,dx}\hat{\phi }_{0}-\frac{\int _{1}^{-1}\left( \hat{\phi }_{1}(x)\phi _{2}(x)\right) \,dx}{\int _{1}^{-1}\left( \hat{\phi }_{1}(x)\hat{\phi }_{1}(x)\right) \,dx}\hat{\phi }_{1}\\ & =  {} \left( -\frac{35}{8}x^{4}+\frac{21}{4}x^{2}-\frac{7}{8}\right) -\frac{{-2}/{5}}{{12}/{5}}\left( -\frac{3}{2}x^{2}+\frac{3}{2}\right) -\frac{0}{{20}/{21}}\left( -\frac{5}{2}x^{3}+\frac{5}{2}x\right) \\ & =  {} -\frac{35}{8}x^{4}+5x^{2}-\frac{5}{8}.\end{aligned}$$

and

$$\begin{aligned} \hat{\phi }_{3} & =  {} \phi _{3}-\frac{\left\langle \hat{\phi }_{0},\phi _{3}\right\rangle }{\left\langle \hat{\phi }_{0},\hat{\phi }_{0}\right\rangle }\hat{\phi }_{0}-\frac{\left\langle \hat{\phi }_{1},\phi _{3}\right\rangle }{\left\langle \hat{\phi }_{1},\hat{\phi }_{1}\right\rangle }\hat{\phi }_{1}- \frac{\left\langle \hat{\phi }_{2},\phi _{3}\right\rangle }{\left\langle \hat{\phi }_{2},\hat{\phi }_{2}\right\rangle }\hat{\phi }_{2}\\ & =  {} \phi _{3}-\frac{\int _{1}^{-1}\left( \hat{\phi }_{0}(x)\phi _{3}(x)\right) \,dx}{\int _{1}^{-1}\left( \hat{\phi }_{0}(x)\hat{\phi }_{0}(x)\right) \,dx}\hat{\phi }_{0}-\frac{\int _{1}^{-1}\left( \hat{\phi }_{1}(x)\phi _{3}(x)\right) \,dx}{\int _{1}^{-1}\left( \hat{\phi }_{1}(x)\hat{\phi }_{1}(x)\right) dx}\hat{\phi }_{1}-\frac{\int _{1}^{-1}\left( \hat{\phi }_{2}(x)\phi _{3}(x)\right) \,dx}{\int _{1}^{-1}\left( \hat{\phi }_{2}(x)\hat{\phi }_{2}(x)\right) dx}\hat{\phi }_{2}\\ & =  {} \left( -\frac{63}{8}x^{5}+\frac{45}{4}x^{3}-\frac{27}{8}x\right) -\frac{0}{{12}/{5}}\left( -\frac{3}{2}x^{2}+\frac{3}{2}\right) -\frac{{-2}/{7}}{{20}/{21}}\left( -\frac{5}{2}x^{3}+\frac{5}{2}x\right) -\frac{0}{{5}/{9}}\left( -\frac{35}{8}x^{4}+5x^{2}-\frac{5}{8}\right) \\ & =  {} -\frac{63}{8}x^{5}+\frac{21}{3}x^{3}-\frac{21}{8}x. \end{aligned}$$

**Fig. 2:**
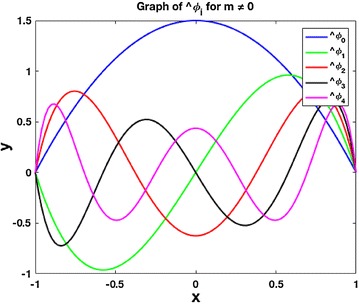
Graphs of $$\hat{\phi }_i$$, $$i=0,1,2,3,4$$

The graphs of the first several members of the sequence $\{ \hat{\phi }_i \}$ are shown in Fig. 2.

Again, we will compare $\phi _{2}$ with $\hat{\phi }_{2}$ and $\phi _{3}$ with $\hat{\phi }_{3}$ to find a general formula for the values of $\hat{\phi _{i}.}$ We obtain the following formula

37 $$\begin{aligned} \hat{\phi }_{2}-\phi _{2} & =  {} -\frac{35}{8}x^{4}+5x^{2}-\frac{5}{8}-\left( -\frac{35}{8}x^{4}+\frac{21}{4}x-\frac{7}{8}\right) \nonumber \\ & =  {} -\frac{1}{4}x^{2}+\frac{1}{4}\nonumber \\ & =  {} \frac{1}{6}\left( -\frac{3}{2}x^{2}+\frac{3}{2}\right) \nonumber \\ & =  {} \frac{1}{6}\hat{\phi }_{0}. \end{aligned}$$

and

$$\begin{aligned} \hat{\phi }_{3}-\phi _{3} & =  {} -\frac{63}{8}x^{5}+\frac{21}{2}x^{3}-\frac{21}{8}x-\left( -\frac{63}{8}x^{5}+\frac{45}{4}x^{3}-\frac{27}{8}x\right) \\ & =  {} -\frac{3}{4}x^{3}+\frac{3}{4}x\\ & =  {} \frac{3}{10}\left( -\frac{5}{2}x^{3}+\frac{5}{2}x\right) \\ & =  {} \frac{3}{10}\hat{\phi }_{1}. \end{aligned}$$

These results suggest a simple recurrence relation for $\hat{\phi }_i$ in terms of $\phi _i$ and $\hat{\phi }_{i-2}$, in which the coefficient of $\hat{\phi }_{i-2}$ is a ratio of triangular numbers $d_i = i(i-1)/[(i+1)(i+2)]$. We therefore define

38 $$\begin{aligned} \hat{\phi }_i = \phi _i - \frac{i(i-1)}{(i+1)(i+2)} \hat{\phi }_{i-2}, \quad i = 2, 3, \ldots , N-2, \end{aligned}$$

with initial conditions

39 $$\begin{aligned} \hat{\phi }_0(x) = \phi _0(x) = 1-x^2, \quad \hat{\phi }_1(x) = \phi _1(x) = \frac{5}{2}(x-x^3). \end{aligned}$$

To prove that these polynomials are actually orthogonal, we first need this result.

### **Lemma 2**

*Let*$\hat{\phi }_j(x)$*be defined as in* (38), (39), *and let*$N_{j}=\left\langle \hat{\phi }_{j},\hat{\phi }_{j}\right\rangle$, $\forall  \quad  j\ge 2.$*Then*

40 $$\begin{aligned} N_{j}=\frac{2\left( j+3\right) \left( j+4\right) }{\left( 2j+5\right) \left( j+2\right) \left( j+1\right) }, \end{aligned}$$

$$\forall  j\ge 2$$ and

$$\begin{aligned} \hat{\phi }_{j}=\phi _{j}+\frac{j\left( j-1\right) }{\left( j+1\right) \left( j+2\right) }\hat{\phi }_{j-2}, \quad  j\ge 2, \quad \hat{\phi }_{j}=\phi _{j}, \quad  j\le 1. \end{aligned}$$

### *Proof*

For the base case, we compute $N_0$ and $N_1$ directly. We have

$$\begin{aligned} N_0 & =  {} \langle \hat{\phi }_0, \hat{\phi }_0 \rangle \\ & =  {} \langle \phi _0, \phi _0 \rangle \\ & =  {} \langle L_0 - L_2, L_0 - L_2 \rangle \\ & =  {} \langle L_0, L_0 \rangle + \langle L_2, L_2 \rangle \\ & =  {} \frac{12}{5}, \end{aligned}$$

and

$$\begin{aligned} N_1 & =  {} \langle \hat{\phi }_1, \hat{\phi }_1 \rangle \\ & =  {} \langle \phi _1, \phi _1 \rangle \\ & =  {} \langle L_1 - L_3, L_1 - L_3 \rangle \\ & =  {} \langle L_1, L_1 \rangle + \langle L_3, L_3 \rangle \\ & =  {} \frac{20}{21}. \end{aligned}$$

For the induction step, we assume there is a $j>2$ such that $N_{j-2}=\frac{2j\left( j+3\right) }{j\left( j+1\right) \left( 2j+3\right) }.$ Now, we must show that the formula (40) is true for *j*. We have

$$\begin{aligned} N_{j}=\left\langle \hat{\phi }_{j},\hat{\phi }_{j}\right\rangle & =  {} \left\langle \phi _{j}+\frac{j\left( j-1\right) }{\left( j+1\right) \left( j+2\right) }\hat{\phi }_{j-2},\phi _{j}+\frac{j\left( j-1\right) }{\left( j+1\right) \left( j+2\right) }\hat{\phi }_{j-2}\right\rangle \\ & =  {} \left\langle \phi _{j},\phi _{j}\right\rangle +\frac{2j\left( j-1\right) }{\left( j+1\right) \left( j+2\right) }\left\langle \phi _{j},\hat{\phi }_{j-2}\right\rangle +\left( \frac{j\left( j-1\right) }{\left( j+1\right) \left( j+2\right) }\right) ^{2}\left\langle \hat{\phi }_{j-2},\hat{\phi }_{j-2}\right\rangle \\ & =  {} \frac{4\left( 2j+3\right) }{\left( 2j+1\right) \left( 2j+5\right) }+\frac{2j\left( j-1\right) }{\left( j+1\right) \left( j+2\right) }\left( -\left\langle L_{j},L_{j}\right\rangle \right) +\left( \frac{j\left( j-1\right) }{\left( j+1\right) \left( j+2\right) }\right) ^{2}N_{j-2}\\ & =  {} \frac{4\left( 2j+3\right) }{\left( 2j+1\right) \left( 2j+5\right) }+\frac{2j\left( j-1\right) }{\left( j+1\right) \left( j+2\right) }\left( \frac{2}{2j+1}\right) +\left( \frac{j\left( j-1\right) }{\left( j+1\right) \left( j+2\right) }\right) ^{2}N_{j-2}\\ & =  {} \frac{24\left( j^{2}+3j+1\right) }{\left( j+1\right) \left( j+2\right) \left( 2j+1\right) \left( 2j+5\right) }+\left( \frac{j\left( j-1\right) }{\left( j+1\right) \left( j+2\right) }\right) ^{2}\left[ \frac{2\left( j+1\right) \left( j+2\right) }{j\left( j-1\right) \left( 2j+1\right) }\right] \\ & =  {} \frac{2\left( j+3\right) \left( j+4\right) }{\left( j+1\right) \left( j+2\right) \left( 2j+1\right) }. \end{aligned}$$

$$\square $$

### **Theorem 5**

*Let*$\hat{\phi }_i$*be obtained by orthogonalizing*$\phi _i$*against*$\hat{\phi }_0, \hat{\phi }_1, \ldots$*Then*$\hat{\phi }_0 = \phi _0$, $\hat{\phi }_1 = \phi _1$, *and*

41 $$\begin{aligned} \hat{\phi }_{j}=\phi _{j}+d_{j}\hat{\phi }_{j-2}, \quad j\ge 2, \end{aligned}$$

*where*$$d_{j}=\frac{j\left( j-1\right) }{\left( j+1\right) \left( j+2\right) }$$.

### *Proof*

For the base case, we first show that $\hat{\phi }_1 = \phi _1$ and $\hat{\phi }_0 = \phi _0$ are already orthogonal. We have

$$\begin{aligned} \left\langle \hat{\phi }_{1},\hat{\phi }_{0}\right\rangle & =  {} \left\langle L_{1}-L_{3},L_{o}-L_{2}\right\rangle \\ & =  {} \left\langle L_{0},L_{1}\right\rangle -\left\langle L_{1},L_{2}\right\rangle -\left\langle L_{0},L_{3}\right\rangle +\left\langle L_{2},L_{3}\right\rangle \\ & =  {} 0. \end{aligned}$$

Next, we show directly that the theorem holds when $j=2$:

42 $$\begin{aligned} \hat{\phi }_{2} & =  {} \phi _{2}-\frac{\left\langle \hat{\phi }_{0},\phi _{2}\right\rangle }{\left\langle \hat{\phi }_{0},\hat{\phi }_{0}\right\rangle }\hat{\phi }_{0}-\frac{\left\langle \hat{\phi }_{1},\phi _{2}\right\rangle }{\left\langle \hat{\phi }_{1},\hat{\phi }_{1}\right\rangle } \hat{\phi }_{1}\nonumber \\ & =  {} -\frac{35}{8}x^{4}-\frac{21}{4}x^{2}-\frac{7}{8}-\frac{-{2}/{5}}{{12}/{5}}\left( -\frac{3}{2}x^{2}+\frac{3}{2}\right) -\frac{0}{{20}/{21}}\left( -\frac{5}{2}x^{3}+\frac{5}{2}x\right) \nonumber \\ & =  {} \phi _{2}+\frac{1}{6}\hat{\phi }_{0}. \end{aligned}$$

For the induction step, we assume that $\hat{\phi }_{0},\ldots ,\hat{\phi }_{j-1}$ are all orthogonal, where $j\ge 2$, and that

$$\begin{aligned} \hat{\phi }_{j}=\phi _{j}+d_{j}\hat{\phi }_{j-2}, \end{aligned}$$

where $$d_{j}=\frac{j(j-1)}{(j+1)(j+2)}$$. Then

$$\begin{aligned} \hat{\phi }_{j+1} & =  {} \phi _{j+1}+\sum _{k=0}^{j}\frac{\left\langle \hat{\phi }_{k},\phi _{j+1}\right\rangle }{\left\langle \hat{\phi }_{k},\hat{\phi }_{k}\right\rangle }\hat{\phi }_{k}\\ & =  {} \phi _{j+1}-\frac{\left\langle \hat{\phi }_{j-1},L_{j+1}\right\rangle -\left\langle \hat{\phi }_{j-1}, L_{j+3}\right\rangle }{\left\langle \hat{\phi }_{j-1},\hat{\phi }_{j-1}\right\rangle }\hat{\phi }_{j-1}\\ & =  {} \phi _{j+1}-\frac{\left\langle \phi _{j-1}+c_{j-1}\hat{\phi }_{j-3},L_{j+1}\right\rangle }{\left\langle \hat{\phi }_{j-1},\hat{\phi }_{j-1}\right\rangle }\hat{\phi }_{j-1}\\ & =  {} \phi _{j+1}-\frac{\left\langle \phi _{j-1},L_{j+1}\right\rangle +c_{j-1}\left\langle \hat{\phi }_{j-3},L_{j+1}\right\rangle }{\left\langle \hat{\phi }_{j-1},\hat{\phi }_{j-1}\right\rangle }\hat{\phi }_{j-1}\\ & =  {} \phi _{j+1}-\frac{\left\langle \phi _{j-1},L_{j+1}\right\rangle }{\left\langle \hat{\phi }_{j-1},\hat{\phi }_{j-1}\right\rangle }\hat{\phi }_{j-1}\\ & =  {} \phi _{j+1}-\frac{\left\langle L_{j-1}-L_{j+1},L_{j+1}\right\rangle }{\left\langle \hat{\phi }_{j-1},\hat{\phi }_{j-1}\right\rangle }\hat{\phi }_{j-1}\\ & =  {} \phi _{j+1}+\frac{\left\langle L_{j+1},L_{j+1}\right\rangle }{\left\langle \hat{\phi }_{j-1},\hat{\phi }_{j-1}\right\rangle }\hat{\phi }_{j-1}\\ & =  {} \phi _{j+1}+\frac{2}{2j+3}\frac{1}{\left\langle \hat{\phi }_{j-1},\hat{\phi }_{j-1}\right\rangle }\hat{\phi }_{j-1}\end{aligned}$$

Using Lemma 2, we obtain

$$\begin{aligned} \hat{\phi }_{j+1} & =  {} \phi _{j+1}+\frac{2}{2j+3}\frac{j\left( j+1\right) \left( 2j+3\right) }{2\left( j+2\right) \left( j+3\right) }\hat{\phi }_{j-1}\\ & =  {} \phi _{j+1}+\frac{j\left( j+1\right) }{\left( j+2\right) \left( j+3\right) }\hat{\phi }_{j-1}. \quad  \end{aligned}$$

$$\square $$

We now confirm that the polynomials defined using the recurrence (41) are orthogonal.

### **Theorem 6**

*Let*$\hat{\phi }_{k}$*be defined as follows*:

$$\begin{aligned} \hat{\phi }_{k}=\phi _{k}+\frac{k\left( k-1\right) }{\left( k+1\right) \left( k+2\right) }\hat{\phi }_{k-2}, \quad  k\ge 2, \quad \hat{\phi }_{k}=\phi _{k}, \quad  k\le 1. \end{aligned}$$

*Then*$$\left\langle \hat{\phi }_{k},\hat{\phi }_{j}\right\rangle =0$$*for*$$j\ne k$$.

### *Proof*

We will show that for each $$k\ge 0,$$$$\left\langle \hat{\phi }_{k},\hat{\phi }_{j}\right\rangle =0$$ for $$0\le j<k.$$ The case $$k=1$$ was handled in the proof of Theorem 5. Proceeding by induction, we assume $$\hat{\phi }_{0},\ldots ,\hat{\phi }_{k-1}$$ are all orthogonal, and show that $$\left\langle \hat{\phi }_{k},\hat{\phi }_{j}\right\rangle =0$$ for $$j=0,1,\ldots ,k-1.$$

Case 1: $$j<k-2$$

$$\begin{aligned} \left\langle \hat{\phi }_{k},\hat{\phi }_{j}\right\rangle & =  {} \left\langle \phi _{k},\hat{\phi }_{j}\right\rangle +\frac{k\left( k-1\right) }{\left( k+1\right) \left( k+2\right) }\left\langle \hat{\phi }_{k-2},\hat{\phi }_{j}\right\rangle \\ & =  {} \left\langle L_{k}-L_{k+2},\hat{\phi }_{j}\right\rangle \\ & =  {} 0. \end{aligned}$$

Case 2: $$j=k-2$$

$$\begin{aligned} \left\langle \hat{\phi }_{k},\hat{\phi }_{j}\right\rangle & =  {} \left\langle \phi _{k},\hat{\phi }_{j}\right\rangle +\frac{k\left( k-1\right) }{\left( k+1\right) \left( k+2\right) }\left\langle \hat{\phi }_{k-2},\hat{\phi }_{j}\right\rangle \\ & =  {} \left\langle L_{k}-L_{k+2},\hat{\phi }_{k-2}\right\rangle +\frac{k\left( k-1\right) }{\left( k+1\right) \left( k+2\right) }\left\langle \hat{\phi }_{k-2},\hat{\phi }_{k-2}\right\rangle \\ & =  {} \left\langle L_{k},\hat{\phi }_{k-2}\right\rangle +\frac{k\left( k-1\right) }{\left( k+1\right) \left( k+2\right) }\left\langle \hat{\phi }_{k-2},\hat{\phi }_{k-2}\right\rangle \\ & =  {} -\left\langle L_{k},L_{k}\right\rangle +\frac{k\left( k-1\right) }{\left( k+1\right) \left( k+2\right) }\left\langle \hat{\phi }_{k-2},\hat{\phi }_{k-2}\right\rangle \\ & =  {} -\frac{2}{2k+1}+\frac{k\left( k-1\right) }{\left( k+1\right) \left( k+2\right) }\left( \frac{2\left( k+1\right) \left( k+2\right) }{k\left( k-1\right) \left( 2k+1\right) }\right) \\ & =  {} 0. \end{aligned}$$

Case 3: $j=k-1$. If $k \ge 3$, then we have

$$\begin{aligned} \left\langle \hat{\phi }_{k},\hat{\phi }_{j}\right\rangle & =  {} \left\langle \phi _{k},\hat{\phi }_{j}\right\rangle +\frac{k\left( k-1\right) }{\left( k+1\right) \left( k+2\right) }\left\langle \hat{\phi }_{k-2},\hat{\phi }_{j}\right\rangle \\ & =  {} \left\langle \phi _{k},\hat{\phi }_{k-1}\right\rangle +\frac{k\left( k-1\right) }{\left( k+1\right) \left( k+2\right) }\left\langle \hat{\phi }_{k-2},\hat{\phi }_{k-1}\right\rangle \\ & =  {} \left\langle L_{k}-L_{k+2},\hat{\phi }_{k-1}\right\rangle \\ & =  {} \left\langle L_{k},\hat{\phi }_{k-1}\right\rangle \\ & =  {} \left\langle L_k, L_{k-1} - L_{k+1} + \frac{(k-1)(k-2)}{k(k+1)} \hat{\phi }_{k-3} \right\rangle \\ & =  {} 0. \end{aligned}$$

If $k=2$, then the steps are the same, except that the term with $\hat{\phi }_{k-3}$ is not present. $\square$

Like all families of orthogonal polynomials, the $\{ \hat{\phi }_k \}$ satisfy the recurrence relation

43 $$\begin{aligned} \beta _j \hat{\phi }_{j+1}(x) = (x - \alpha _j)\hat{\phi }_j(x) - \gamma _{j-1} \hat{\phi }_{j-1}(x). \end{aligned}$$

By analogy with (21), (22) and (23), we have

44 $$\begin{aligned} \alpha _j & =  {} \frac{\langle \hat{\phi }_j, x\hat{\phi }_j \rangle }{\langle \hat{\phi }_j, \hat{\phi }_j \rangle }, \end{aligned}$$

45 $$\begin{aligned} \beta _j & =  {} \frac{\langle \hat{\phi }_{j+1}, x\hat{\phi }_j \rangle }{\langle \hat{\phi }_{j+1}, \hat{\phi }_{j+1} \rangle }, \end{aligned}$$

46 $$\begin{aligned} \gamma _j & =  {} \frac{\langle \hat{\phi }_{j+1}, x\hat{\phi }_j \rangle }{\langle \hat{\phi }_j, \hat{\phi }_j \rangle }, . \end{aligned}$$

Because $\hat{\phi }_j$ contains only terms of odd degree if *j* is odd and of even degree if *j* is even, just like the Legendre polynomials, it is easily shown that $\alpha _j = 0$ for $j=1,2,\ldots$ We will now find the values of $\beta _{j}$ and $\gamma _j$.

### **Theorem 7**

*Let*$\beta _j$*be defined as in* (45). *Then*$\beta _{j}=\frac{j+3}{2j+5},\quad \forall j\ge 0.$

### *Proof*

We show the base case $j=0$ directly:

$$\begin{aligned} \beta _{0} & =  {} \frac{\left\langle \hat{\phi }_{1},x\hat{\phi }_{0}\right\rangle }{\left\langle \hat{\phi }_{1},\hat{\phi }_{1}\right\rangle }\\ & =  {} \frac{\int _{-1}^{1}\hat{\phi }_{1}(x) x\hat{\phi }_{0}(x)\,dx}{\int _{-1}^{1}\hat{\phi }_{1}(x) \hat{\phi }_{1}(x)\,dx}\\ & =  {} \frac{3}{5}. \end{aligned}$$

For the induction step, we assume there is a $j\ge 0$ such that $\beta _{j-1}=\frac{j+2}{2j+3}$.

Then, using (45), we have $\beta _{j}=\frac{\left\langle \hat{\phi }_{j+1},x\hat{\phi }_{j}\right\rangle }{\left\langle \hat{\phi }_{j+1},\hat{\phi }_{j+1}\right\rangle }$ and $\hat{\phi }_{j}=\phi _{j}+d_{j}\hat{\phi }_{j-2}$ where $d_{j}=\frac{j\left( j-1\right) }{\left( j+1\right) \left( j+2\right) }.$ For the numerator, we have

47 $$\begin{aligned} \left\langle \hat{\phi }_{j+1},x\hat{\phi }_{j}\right\rangle & =  {} \left\langle \phi _{j+1}+d_{j+1}\hat{\phi }_{j},x\left( \phi _{j}+d_{j}\hat{\phi }_{j-2}\right) \right\rangle \nonumber \\ & =  {} \left\langle \phi _{j+1},x\phi _{j}\right\rangle +d_{j}\left\langle \hat{\phi }_{j-2},x\phi _{j+1}\right\rangle +d_{j+1}\left\langle \hat{\phi }_{j-1},x\phi _{j}\right\rangle \nonumber \\& \quad +d_{j}d_{j+1}\left\langle \hat{\phi }_{j-1},x\hat{\phi }_{j-2}\right\rangle . \end{aligned}$$

We now compute each part of this numerator as follows:

48 $$\begin{aligned} \left\langle \phi _{j+1},x\phi _{j}\right\rangle & =  {} \left\langle L_{j+1}-L_{j+3},x\left( L_{j}-L_{j+2}\right) \right\rangle \nonumber \\ & =  {} \left\langle L_{j+1}-L_{j+3},\left( \frac{j+1}{2j+1}L_{j+1}+\frac{j}{2j+1}L_{j-1}\right) -\left( \frac{j+3}{2j+5}L_{j+3}+\frac{j+2}{2j+5}L_{j+1}\right) \right\rangle \nonumber \\ & =  {} \frac{j+1}{2j+1}\left\langle L_{j+1},L_{j+1}\right\rangle -\frac{j+2}{2j+5}\left\langle L_{j+1},L_{j+1}\right\rangle +\frac{j+3}{2j+5}\left\langle L_{j+3},L_{j+3}\right\rangle \nonumber \\ & =  {} \frac{j+1}{2j+1}\left( \frac{2}{2j+3}\right) -\frac{j+2}{2j+5}\left( \frac{2}{2j+3}\right) +\frac{j+3}{2j+5}\left( \frac{2}{2j+7}\right) \nonumber \\ & =  {} \frac{2(j+2)}{(2j+1)\left( 2j+7\right) },\nonumber \\ \left\langle \hat{\phi }_{j-2},x\phi _{j+1}\right\rangle & =  {} \left\langle \hat{\phi }_{j-2},\left( \frac{j+2}{2j+3}L_{j+2}+\frac{j+1}{2j+3}L_{j}\right) -\left( \frac{j+4}{2j+7}L_{j+4}+\frac{j+3}{2j+7}L_{j+2}\right) \right\rangle \nonumber \\& =  {} \frac{j+1}{2j+3}\left\langle \hat{\phi }_{j-2},L_{j}\right\rangle \nonumber \\& = {} -\frac{j+1}{2j+3}\left\langle L_{j},L_{j}\right\rangle \nonumber \\& = {} -\frac{j+1}{2j+3}\left( \frac{2}{2j+1}\right) \nonumber \\& = {} -\frac{2\left( j+1\right) }{\left( 2j+1\right) \left( 2j+3\right) }. \end{aligned}$$

Then

49 $$\begin{aligned} d_{j}\left\langle \hat{\phi }_{j-2},x\phi _{j+1}\right\rangle & = {} \left( \frac{j\left( j-1\right) }{\left( j+1\right) \left( j+2\right) }\right) \left( -\frac{2\left( j+1\right) }{\left( 2j+1\right) \left( 2j+3\right) }\right) \nonumber \\& = {} \frac{-2j\left( j-1\right) }{\left( j+2\right) \left( 2j+1\right) \left( 2j+3\right) }. \end{aligned}$$

For the third term in (47), we have

$$\begin{aligned} \left\langle \hat{\phi }_{j-1},x\phi _{j}\right\rangle & = {} \left\langle \hat{\phi }_{j-1},\left( \frac{j+1}{2j+1}L_{j+1}+\frac{j}{2j+1}L_{j-1}\right) -\left( \frac{j+3}{2j+5}L_{j+3}+\frac{j+2}{2j+5}L_{j+1}\right) \right\rangle \\ & = {} \frac{j+1}{2j+1}\left\langle \hat{\phi }_{j-1},L_{j+1}\right\rangle +\frac{j}{2j+1}\left\langle \hat{\phi }_{j-1},L_{j-1}\right\rangle -\frac{j+2}{2j+5}\left\langle \hat{\phi }_{j-1},L_{j+1}\right\rangle \\ & = {} \frac{j+1}{2j+1}\left( -\left\langle L_{j+1},L_{j+1}\right\rangle \right) +\frac{j}{2j+1}\left[ \left( 1-d_{j-1}\right) \left\langle L_{j-1},L_{j-1}\right\rangle \right] \\&-\frac{j+2}{2j+5}\left[ -\left\langle L_{j+1},L_{j+1}\right\rangle \right] \\ & = {} -\frac{j+1}{2j+1}\left( \frac{2}{2j+3}\right) +\frac{j}{2j+1}\left( \frac{4}{j\left( j+1\right) }\right) -\frac{j+2}{2j+5}\left( \frac{2}{2j+3}\right) \\ & = {} \frac{6\left( j+3\right) }{\left( j+1\right) \left( 2j+1\right) \left( 2j+5\right) }, \end{aligned}$$

and therefore

50 $$\begin{aligned} d_{j+1}\left\langle \hat{\phi }_{j-1},x\phi _{j}\right\rangle & = {} \frac{j\left( j+1\right) }{\left( j+2\right) \left( j+3\right) }\left( \frac{6\left( j+3\right) }{\left( j+1\right) \left( 2j+1\right) \left( 2j+5\right) }.\right) \nonumber \\ & = {} \frac{6j}{\left( j+2\right) \left( 2j+1\right) \left( 2j+5\right) }. \end{aligned}$$

We rearrange the formula for $\beta _{j-2}$ to obtain the following:

$$\begin{aligned} \left\langle \hat{\phi }_{j-1},x\hat{\phi }_{j-2}\right\rangle & = {} \beta _{j-2}\left\langle \hat{\phi }_{j-1},\hat{\phi }_{j-1}\right\rangle \\ & = {} \frac{j+1}{2j+1}\frac{2(\left( j+2\right) \left( j+3\right) }{j\left( j+1\right) \left( 2j+3\right) }\\ & = {} \frac{2\left( j+2\right) \left( j+3\right) }{j\left( 2j+1\right) \left( 2j+3\right) }. \end{aligned}$$

Therefore,

51 $$\begin{aligned} d_{j}d_{j+1}\left\langle \hat{\phi }_{j-1},x\hat{\phi }_{j-2}\right\rangle & = {} \left( \frac{j\left( j-1\right) }{\left( j+1\right) \left( j+2\right) }\right) \left( \frac{j\left( j+1\right) }{\left( j+2\right) \left( j+3\right) }\right) \left( \frac{2\left( j+2\right) \left( j+3\right) }{j\left( 2j+1\right) \left( 2j+3\right) }\right) \nonumber \\ & = {} \frac{2j\left( j-1\right) }{\left( j+1\right) \left( 2j+1\right) \left( 2j+3\right) }. \end{aligned}$$

Now we can use the results from Eqs. (48)–(51) to determine the numerator of $\beta _{j}.$

52 $$\begin{aligned} \left\langle \hat{\phi }_{j+1},x\hat{\phi }_{j}\right\rangle & = {} \frac{2(j+2)}{(2j+1)\left( 2j+7\right) }-\frac{2j\left( j-1\right) }{\left( j+2\right) \left( 2j+1\right) \left( 2j+3\right) }+\frac{6j}{\left( j+2\right) \left( 2j+1\right) \left( 2j+5\right) }\nonumber \\& \quad + \frac{2j\left( j-1\right) }{\left( j+1\right) \left( 2j+1\right) \left( 2j+3\right) } \nonumber \\ & = {} \frac{2\left( j+4\right) \left( j+5\right) }{\left( j+2\right) \left( 2j+5\right) \left( 2j+7\right) } \end{aligned}$$

Thus,

$$\begin{aligned} \beta _{j} & = {} \frac{2\left( j+4\right) \left( j+5\right) }{\left( j+2\right) \left( 2j+5\right) \left( 2j+7\right) }\frac{\left( j+2\right) \left( j+3\right) \left( 2j+7\right) }{2\left( j+4\right) \left( j+5\right) }\\ & = {} \frac{j+3}{2j+5}.  \quad  \end{aligned}$$

$$\square $$

From (46), (52), and Lemma 2, we obtain

53 $$\begin{aligned} \gamma _j & = {} \frac{2\left( j+4\right) \left( j+5\right) }{\left( j+2\right) \left( 2j+5\right) \left( 2j+7\right) } \frac{\left( j+1\right) \left( j+2\right) \left( 2j+5\right) }{2\left( j+3\right) \left( j+4\right) } \nonumber \\ & = {} \frac{(j+1)\left( j+5\right) }{(j+3)\left( 2j+7\right) }. \end{aligned}$$

In summary, we have

54 $$\begin{aligned} \frac{j+3}{2j+5} \hat{\phi }_{j+1}(x) = x \hat{\phi }_j(x) - \frac{j\left( j+4\right) }{(j+2)\left( 2j+5\right) } \hat{\phi }_{j-1}(x). \end{aligned}$$

Equation (41) can be rewritten as $\phi _{j}=\hat{\phi }_{j}-d_{j}\hat{\phi }_{j-2}$. Now, we have the system

55 $$\begin{aligned} \Phi =\hat{\Phi }D, \quad D = \left[ \begin{array}{ccccccc} 1 &{} 0 &{} -d_{2}\\ &{} 1&{} 0 &{} -d_{3}\\ &{} &{} 1 &{} 0 &{}\ddots \\ &{} &{} &{} 1 &{}\ddots &{}-d_{n} \\ &{} &{} &{} &{}\ddots &{} 0\\ &{} &{} &{} &{} &{} 1\end{array}\right] \end{aligned}$$

where $$ \Phi =\left[ \begin{array}{cccc} \phi _{0}(\mathbf {x})&\phi _{1}(\mathbf {x})&\cdots&\phi _{n}(\mathbf {x}) \end{array}\right] $$ and $$\hat{\Phi }=\left[ \begin{array}{cccc}\hat{\phi }_{0}(\mathbf {x})&\hat{\phi }_{1}(\mathbf {x})&\cdots&\hat{\phi }_{n}(\mathbf {x})\end{array}\right] $$, with $\mathbf{x}$ being a vector of at least $n+3$ Legendre-Gauss-Lobatto points. This ensures that the columns of $\hat{\Phi }$ are orthogonal.

Then, given $f \in X_{n+2}(m)$, we can obtain the coefficients $\tilde{f}_i$ in

$$\begin{aligned} f(x) = \sum _{i=0}^n \hat{f}_i \hat{\phi }_i(x) \end{aligned}$$

by simply computing $\hat{f}_i = \langle \hat{\phi }_i, f \rangle / N_i$, where $N_i$ is as defined in (40). Then the coefficients $f_i$ in

$$\begin{aligned} f(x) = \sum _{i=0}^n f_i \phi _i(x) \end{aligned}$$

can be obtained by solving the system $D\mathbf{f} = \hat{\mathbf{f}}$ using back substitution, where *D* is as defined in (55). These coefficients can be used in conjunction with the discretization used in Shen (1997), which makes use of the basis $\{ \phi _i \}$.

## Recurrence relations for generalized jacobi polynomials

The families of orthogonal polynomials developed in the preceding two sections are orthogonal with respect to the weight function $\omega (x)\equiv 1$. In Guo et al. (2009), Shen (2003), families of generalized Jacobi polynomials/functions (GJP/Fs) are defined in such a way as to satisfy specified boundary conditions, such as the ones employed in this paper. These functions are orthogonal with respect to a weight function that is determined by the boundary conditions. However, it can be seen from (10) that an alternative weight function may be preferable when solving certain PDEs. In this section, we discuss the modification of sequences of orthonormal polynomials and their three-term recurrence relations as a consequence of changes in the underlying weight function.

Let $J_n$ be the $n\times n$ Jacobi matrix consisting of the recursion coefficients corresponding to a sequence of polynomials $p_j(t)$, $j=0,1,\ldots , n-1$ that is orthonormal with respect to the inner product

$$\begin{aligned} \langle f, g \rangle _\omega = \int _{-1}^1 \overline{f(t)}g(t)\,d\lambda (t), \end{aligned}$$

where $d\lambda (t) = \omega (t)\,dt$, and let $\tilde{J}_n$ be the $n\times n$ Jacobi matrix for a sequence of polynomials $\tilde{p}_j(t)$, $j=0,1,\ldots , n-1$ that is orthonormal with respect to the inner product

$$\begin{aligned} \langle f,g \rangle _{\tilde{\omega }} = \int _{-1}^1 \overline{f(t)}g(t)\,d\tilde{\lambda }(t), \end{aligned}$$

where the measure $d\tilde{\lambda }(t) = \tilde{\omega }(t)\,dt$ is a modification of $d\lambda (t)$ by some factor. The following procedures can be used to generate $\tilde{J}_n$ from $J_n$:

- *Multiplying by a linear factor:* In the case $d\tilde{\lambda }(t) = (t-c)\,d\lambda (t)$, we have
  56 $$\begin{aligned} \tilde{J}_n = L^T L + c I + \left( \frac{\delta _{n-1}}{\ell _{nn}} \right) ^2 \mathbf{e}_n \mathbf{e}_n^T, \end{aligned}$$
  where $\omega(J_n - c I) = LL^T$ is the Cholesky factorization (Gautschi 2002; Golub and Kautsky 1983).
- *Dividing by a linear factor:* In the case $d\tilde{\lambda }(t) = (t-c)^{-1}\, d\lambda (t)$, where *c* is near or on the boundary of the interval of integration, the inverse Cholesky (IC) procedure (Elhay and Kautsky 1994) can be used to obtain $\tilde{J}_n$. We have
  $$\begin{aligned} \tilde{J}_n = L^{-1} J_n L - cI + \frac{\delta _{n-1}}{\ell _{nn}} \mathbf{e}_n \mathbf{c}^T, \end{aligned}$$
  where $I = (J_n - cI)LL^T + \mathbf{e}_n \mathbf{d}^T$ and $\mathbf{c}$ and $\mathbf{d}$ are vectors that need not be computed if one is content with only computing $\tilde{J}_{n-1}$.

In either case, the original and modified polynomials are related by *L*:

$$\begin{aligned} \mathbf{p}(t) = L\tilde{\mathbf{p}}(t), \end{aligned}$$

where $$\mathbf{p}(t) = \left[ \begin{array}{ccc} p_0(t)&\ldots&p_{n-1}(t) \end{array} \right] ^T$$ and $$\tilde{\mathbf{p}}(t) = \left[ \begin{array}{ccc} \tilde{p}_0(t)&\ldots&\tilde{p}_{n-1}(t) \end{array} \right] ^T$$.

While three-term recurrence relations for the Jacobi polynomials are well-known, we are not aware of similar recurrence relations for GJPs. We now present efficient algorithms for modifying either of the families of polynomials $\{ \tilde{\phi }_j \}$, $\{ \hat{\phi }_j \}$ to obtain such recurrences.

### Boundary condition $p(1)=0$

We first demonstrate how the polynomials $\{ \tilde{\phi }_j \}$ from section “The case *m* = 0” can be modified to obtain the three-term recurrence for the GJPs

57 $$\begin{aligned} \varphi _j(x) = (1-x)J_{j}^{1,0}(x) = \frac{(-1)^{j}}{2^{j}(j)!} \frac{d^{j}}{dx^{j}}\left\{ (1-x)^{j+1}\right\} , \quad j = 0, 1, \ldots , \end{aligned}$$

which are orthogonal on $(-1,1)$ with respect to the weight function $(1-x)^{-1}$ (Guo et al. 2009; Shen 2003). Like the $\{ \tilde{\phi }_j \}$, these polynomials satisfy the boundary condition $\varphi _j(1) = 0$.

Let

58 $$\begin{aligned} J_n = \left[ \begin{array}{ccccc} \alpha _0 &{} \gamma _0 &{} &{} &{} \\ \beta _0 &{} \alpha _1 &{} \gamma _1 &{} &{} \\ &{} \ddots &{} \ddots &{} \ddots &{} \\ &{} &{} \beta _{n-3} &{} \alpha _{n-2} &{} \gamma _{n-2} \\ &{} &{} &{} \beta _{n-2} &{} \alpha _{n-1} \end{array} \right] \end{aligned}$$

be the matrix of recursion coefficients for the $\{ \tilde{\phi }_j \}_{j=0}^{n-1}$, where $\alpha _j$, $\beta _j$ and $\gamma _j$ are as defined in (21), (22), (23), respectively. First, we apply a diagonal similarity transformation to symmetrize $J_n$, which yields

59 $$\begin{aligned} \tilde{J}_n = \left[ \begin{array}{ccccc} \alpha _0 &{} \delta _0 &{} &{} &{} \\ \delta _0 &{} \alpha _1 &{} \delta _1 &{} &{} \\ &{} \ddots &{} \ddots &{} \ddots &{} \\ &{} &{} \delta _{n-3} &{} \alpha _{n-2} &{} \delta _{n-2} \\ &{} &{} &{} \delta _{n-2} &{} \alpha _{n-1} \end{array} \right] \end{aligned}$$

where $$\delta _j = \sqrt{\gamma _j\beta _j}$$ for $$j=0,1,\ldots , n-2$$.

Let $\hat{J}_n$ be the Jacobi matrix for the polynomials $\varphi _j(x)$. Since its measure is a modification of that of $J_n$ and $\tilde{J}_n$ by dividing by a linear factor, certainly the IC algorithm can be used to compute $\hat{J}_{n-1}$ directly from $\tilde{J}_n$, but this requires $O(n^3)$ arithmetic operations, which exceeds the cost of computing the entries of $\hat{J}_n$ directly as inner products using the Rodriguez formula (57).

Alternatively, we can invert the procedure described above for handling modification by multiplying by a linear factor. First, we let $T_n = I - \tilde{J}_n$, in view of the modification $d\tilde{\lambda }(t) = (1-t)^{-1} d\lambda (t)$. Then, we solve the (*n*, *n*) entry of the matrix equation

$$\begin{aligned} T_n = L^T L + \left( \frac{\hat{\delta }_{n-1}}{\ell _{nn}} \right) ^2 \mathbf{e}_n \mathbf{e}_n^T \end{aligned}$$

for $\ell _{nn}^2$, where *L* is lower triangular. As this equation is quadratic in $\ell _{nn}^2$, we choose the larger root. The entry $\hat{\delta }_{n-1}$ of $\hat{J}_n$ can be computed using (57).

Next, we compute the factorization

$$\begin{aligned} T_n - \left( \frac{\hat{\delta }_{n-1}}{\ell _{nn}} \right) ^2 \mathbf{e}_n \mathbf{e}_n^T = L^T L, \end{aligned}$$

which amounts to performing a Cholesky factorization “in reverse”, as reversing the order of the rows and columns of this matrix equation leads to a Cholesky factorization. Finally, we obtain

$$\begin{aligned} \hat{J}_n = I - LL^T. \end{aligned}$$

This matrix actually differs from the correct $\hat{J}_n$ in the (*n*, *n*) entry. Therefore, deleting the last row and column yields the correct $\hat{J}_{n-1}$. The entire procedure can be carried out in only *O*(*n*) arithmetic operations, due to the fact that *L* is actually lower bidiagonal.

### Boundary conditions $p(-1)=p(1)=0$

We now show how to efficiently obtain recursion coefficients for the GJPs

60 $$\begin{aligned} \hat{\varphi }_j(x) = \frac{(-1)^j}{2^j j!} \frac{d^j}{dx^j} \left[ (1-x)^{j+1} (1+x)^{j+1} \right] , \quad j = 0, 1, \ldots \end{aligned}$$

which are orthogonal on $(-1,1)$ with respect to the weight function $(1-x)^{-1}(1+x)^{-1}$. Like the $\{ \hat{\phi }_j \}$ from section “The case *m* ≠ 0”, these polynomials satisfy the boundary conditions $\varphi _j(-1) = \varphi _j(1) = 0$.

Let $J_n$, $\tilde{J}_n$ be defined as in (58), (59), except that $\alpha _j$, $\beta _j$ and $\gamma _j$ are as defined in (44), (45), (46), respectively, and let $\hat{J}_n$ be the Jacobi matrix for the polynomials $\hat{\varphi }_j(x)$. Since its measure is a modification of that of $J_n$ and $\tilde{J}_n$ by dividing by two distinct linear factors, the IC algorithm can be applied twice to compute $\hat{J}_{n-2}$ directly from $\tilde{J}_n$, but as before, we seek a more efficient approach.

The main idea is to apply the process from section “[Sec Sec9]” twice. In this case, however, it is more complicated because we do not have all of the information we need. As an intermediate step, let $\bar{J}_n$ be the Jacobi matrix for polynomials $\bar{\varphi }_j(x)$ that are orthonormal with respect to the weight function $\bar{\omega }(x) = (1-x)^{-1}$. The goal is to first obtain $\bar{J}_{n-1}$ from $\tilde{J}_n$, and then obtain $\hat{J}_{n-2}$ from $\bar{J}_{n-1}$.

As before, we let $T_n = I - \tilde{J}_n$. We then need to solve the (*n*, *n*) entry of the matrix equation

61 $$\begin{aligned} T_n = L^T L + \left( \frac{\bar{\delta }_{n-1}}{\ell _{nn}} \right) ^2 \mathbf{e}_n \mathbf{e}_n^T \end{aligned}$$

for $\ell _{nn}^2$, where $\bar{\delta }_{n-1} = \langle x\bar{\varphi }_{n-2}, \bar{\varphi }_{n-1} \rangle _{\bar{\omega }}$. However, unlike in section “[Sec Sec10]”, the value of $\bar{\delta }_{n-1}$ is unknown. For now, we leave it as a variable and describe the remainder of the procedure.

Proceeding as before, we compute the factorization

$$\begin{aligned} T_n = \left( \frac{\bar{\delta }_{n-1}}{\ell _{nn}} \right) ^2 \mathbf{e}_n \mathbf{e}_n^T = L^T L, \end{aligned}$$

and then obtain $\bar{J}_n = I - LL^T$. As this differs from the true $\bar{J}_n$ in the (*n*, *n*) entry, we delete the last row and column to obtain $\bar{J}_{n-1}$.

To accomplish the modification of the weight function by dividing by $(1+x)$, we can proceed in a similar manner. We set $\bar{T}_{n-1} = I + \bar{J}_{n-1}$, and then solve the $(n-1,n-1)$ entry of the matrix equation

$$\begin{aligned} \bar{T}_{n-1} = \bar{L}^T \bar{L} + \left( \frac{\hat{\delta }_{n-2}}{\ell _{n-1,n-1}} \right) ^2 \mathbf{e}_{n-1} \mathbf{e}_{n-1}^T \end{aligned}$$

for $\ell _{n-1,n-1}^2$, where $\hat{\delta }_{n-2}$ can be computed using (60).

After computing the factorization

$$\begin{aligned} \bar{T}_{n-1} - \left( \frac{\hat{\delta }_{n-2}}{\ell _{n-1,n-1}} \right) ^2 = \bar{L}^T \bar{L} \end{aligned}$$

we finally obtain

$$\begin{aligned} \hat{J}_{n-1} = \bar{L}\bar{L}^T - I, \end{aligned}$$

and delete the last row and column to obtain $\hat{J}_{n-2}$.

To overcome the obstacle that $\bar{\delta }_{n-1}$ is unknown, we note that correct value of the $(n-2,n-2)$ entry of $\hat{J}_{n-2}$ is known; its value can be obtained using (60) but in this case, it can be determined using properties of even and odd functions that its value must be zero. We therefore solve the nonlinear equation

$$\begin{aligned} F(\bar{\delta }_{n-1}) = 0, \end{aligned}$$

where $F(\delta )$ is the $(n-2,n-2)$ entry of $\hat{J}_{n-2}$ obtained from $\tilde{J}_n$ using the above procedure, with $\bar{\delta }_{n-1} = \delta$.

This equation can be solved using various root-finding methods, such as the secant method. By applying the quadratic formula in solving (61), it can be determined that the solution must lie in (0, 1 / 2]. Choosing initial guesses close to the upper bound of 1 / 2 yields rapid convergence. To improve efficiency, it should be noted that it is not necessary to compute any of the matrices in this algorithm in their entirety to obtain the $(n-2,n-2)$ entry of $\hat{J}_{n-2}$; only a select few entries from the lower right corner of each matrix are needed. As such, it is possible to solve for $\bar{\delta }_{n-1}$ in *O*(1) arithmetic operations, and compute $\hat{J}_{n-2}$ in *O*(*n*) operations overall.

## Conclusions

We have obtained recurrence relations for generating orthogonal polynomials on the interval $(-1,1)$ that satisfy the boundary conditions (1) $p(1)=0$ and (2) $p(-1)=p(1)=0$. These families of orthogonal polynomials can be used to easily implement transformation matrices between physical and frequency space for function spaces of interest for solving PDEs in polar and cylindrical geometries.

While these polynomials are orthogonal with respect to the weight function $\omega (s)\equiv 1$, it has been shown that they can easily be modified to be orthogonal with respect to the other weight functions. When modified as such to obtain GJPs, recursion coefficients can be obtained with far greater efficiency than by computing the required inner products directly.

Future work includes the development of numerical methods that make use of these families of orthogonal polynomials, or modifications thereof. This includes the adaptation of Krylov subspace spectral methods (Palchak et al. 2015) to polar and cylindrical geometries (Richardson and Lambers 2017).
